# Catechol-*O*-methyltransferase rs4680 and rs4818 haplotype association with treatment response to olanzapine in patients with schizophrenia

**DOI:** 10.1038/s41598-020-67351-5

**Published:** 2020-06-22

**Authors:** Matea Nikolac Perkovic, Marina Sagud, Maja Zivkovic, Suzana Uzun, Gordana Nedic Erjavec, Oliver Kozumplik, Dubravka Svob Strac, Ninoslav Mimica, Alma Mihaljevic Peles, Nela Pivac

**Affiliations:** 1 Division of Molecular Medicine, Rudjer Boskovic Institute, Bijenicka 54, 10000 Zagreb, Croatia; 2 University Hospital Center Zagreb, Kispaticeva 12, 10000 Zagreb, Croatia; 3 School of Medicine, University of Zagreb, Salata 3, 10000 Zagreb, Croatia; 4 Department of General Psychiatry, University Psychiatric Hospital Vrapce, Bolnicka cesta 32, 10000 Zagreb, Croatia; 5 Faculty of Medicine, Josip Juraj Strossmayer University, Josipa Huttlera 4, 31000 Osijek, Croatia

**Keywords:** Molecular biology, Neuroscience, Biomarkers, Diseases, Molecular medicine

## Abstract

Antipsychotic drugs target primarily dopaminergic system which makes catechol-*O*-methyltransferase (COMT) an interesting target in studies searching for treatment response predictors in schizophrenia. The study assessed the association of the *COMT* rs4680 and rs4818 polymorphisms with therapeutic response to olanzapine, risperidone, clozapine or other antipsychotic medication after 8 weeks of monotherapy in patients with schizophrenia. 521 Caucasian patients with schizophrenia received a monotherapy with olanzapine (10–20 mg/day; N = 190), risperidone (3–6 mg/day; N = 99), or clozapine (100–500 mg/day; N = 102). The fourth group (N = 130) consisted of patients receiving haloperidol (3–15 mg/day), fluphenazine (4–25 mg/day) or quetiapine (50–800 mg/day). Treatment response was defined as a 50% reduction from the baseline positive and negative syndrome scale (PANSS) total and subscale scores, but also as an observed percentage reduction from the initial PANSS_0–6_ total and subscale scores. Carriers of the *COMT* rs4680 A allele and carriers of the *COMT* rs4680–rs4818 C-A haplotype block had greater reduction in the PANSS total scores following olanzapine treatment, compared to carriers of the *COMT* rs4680 GG genotype and other *COMT* rs4680–rs4818 haplotypes. The *COMT* rs4680 A allele, and *COMT* rs4680–rs4818 C-A haplotype, were significantly associated with therapeutic response in patients treated with olanzapine, but not in patients treated with other antipsychotics.

## Introduction

All antipsychotic drugs target primarily dopamine receptors^1^, and, therefore, the genes coding for components of dopaminergic system are the candidate genes that have been studied as valid predictors of treatment response to antipsychotics in schizophrenia. Catechol-*O*-methyltransferase (COMT), an enzyme responsible for methylation of catecholamines (dopamine, epinephrine, and norepinephrine), regulates dopamine degradation and therefore impacts prefrontal dopaminergic function. There are numerous polymorphisms of the *COMT* gene, but the most frequently studied is a functional polymorphism Val158Met (rs4680) that affects enzyme activity^2–4^, and rs4818 polymorphism that affects *COMT* expression^5^. The *COMT* Val158Met (rs4680) has been investigated extensively as a possible genetic marker of treatment response^1, 4^, treatment resistance^7, 8^, or symptomatic remission^9^ in schizophrenia, but its role is still not clear^1, 10^. *COMT* rs4680 polymorphism, a G/A substitution, results in the amino acid change from valine (Val) to methionine (Met) at codon 158 of membrane bound COMT (MB-COMT) and at codon 108 of soluble short form (S-COMT), and it leads to three or fourfold decrease in the enzymatic activity in the A (Met) carriers. Favorable response to antipsychotics was detected in patients, carriers of the *COMT* rs4680 AA (Met/Met) genotype in a meta-analysis^1^, whereas over-representation of the G (Val) allele was found in poor responders with schizophrenia^11^. On the other hand, several studies did not confirm the significant association between *COMT* rs4680 and treatment response to olanzapine or other typical and atypical antipsychotics^12–16^, or with remission in schizophrenia^9^. *COMT* rs4818 polymorphism, located in exon 4, is a synonymous polymorphism, consisting of a C/G substitution at codon 86 of the S-COMT and codon 136 of the MB-COMT, corresponding to a leucine residue. This polymorphism affects prefrontal dopamine function^5^. Carriers of the *COMT* rs4818 GG genotype have higher COMT activity than CC genotype carriers, and the presence of the G allele leads to reduced tonic prefrontal cortex dopamine signaling^5^. The *COMT* rs4680–rs4818 C-A haplotype has been associated with treatment response^11^, but also with treatment resistance^7^ in schizophrenia. *COMT* rs4818 polymorphism was also associated, in a haplotype analysis, with a treatment response to antidepressant medication in patients with depression^17^. Namely, regrading response to antidepressants, the *COMT* haplotype C-C-A (rs4633–rs4818–rs4680) was more frequent in the responders compared to non-responders^17^. These results suggest that, in addition to genotype- or allele-based approaches, haplotype-based association studies are also powerful tools in evaluating genetic underpinnings of treatment response in schizophrenia^7, 11^.

In our recent study, which used criteria for remission defined as a reduction to mild levels on the key 8 symptoms on the Positive and Negative Syndrome Scale (PANSS) (items P1, P2, P3, N1, N4, N6, G5, G9) for at least 6 months^18^, carriers of the *COMT* rs4680 GA genotype have shown a trend for achieving symptomatic remission^9^. Literature data reviewed in a meta-analysis suggest that carriers of one or two *COMT* rs4680 A alleles show better response than G allele carriers to treatment with atypical antipsychotics^1^. Haplotype data revealed that the presence of the C-A haplotype (rs4680–rs4818) is related to better response^11^. Therefore, in this longitudinal study, which evaluated treatment response, and not treatment resistance or remission, we expected that *COMT* rs4680 A allele, *COMT* rs4818 C allele, and C-A (rs4680–rs4818) haplotype, will be more frequently represented among patients with schizophrenia with a good response to treatment. There are contradictory and inconsistent data on the association between *COMT* genotype and haplotype variants and treatment response to different antipsychotics in schizophrenia, and criteria for the treatment response vary significantly^19^. Therefore, the aim of this study was to evaluate genotype- and haplotype-based association of the *COMT* rs4680 and rs4818 with the much better treatment response^19^ to monotherapy with olanzapine, risperidone, clozapine, or merged group treated with haloperidol, fluphenazine, or quetiapine, in ethnically homogeneous Caucasian subjects with chronic schizophrenia of both sexes.

## Results

Significant differences (Kruskal–Wallis ANOVA followed by the Dunn post hoc test) were detected in age, PANSS total, positive, negative, and general psychopathology scores at baseline and after 8 weeks of treatment with specific antipsychotic between the group of patients treated with olanzapine, risperidone, clozapine or other antipsychotics (Table 1). Patients treated with olanzapine were significantly younger than the other patients (p = 0.005). Patients treated with olanzapine, risperidone or other antipsychotics had marginally to significantly lower (p = 0.014–0.001) total PANSS scores and PANSS positive and negative subscale scores at baseline and after 8 weeks of treatment (p = 0.006–0.001), compared to patients treated with clozapine (Table 1). At baseline, no significant differences (p = 0.045) were found in patients treated with clozapine, compared to patients treated with olanzapine, risperidone, and other antipsychotics, while after treatment, nominally higher (p = 0.013) PANSS general psychopathology scores were detected in patients receiving clozapine therapy (Table 1). There were no differences between the groups of patients treated with different antipsychotics in terms of disease duration and number of episodes (Table 1). However, patients treated with olanzapine were more frequently smokers (p < 0.001) then patients treated with other antipsychotics (Table 1).

**Table 1:** Demographic and clinical data of 521 schizophrenic patients treated with olanzapine, risperidone, clozapine and other antipsychotics.

	Olanzapine *n* = 190	Risperidone *n* = 99	Clozapine *n* = 102	Other antipsychotics *n* = 130	Test statistics	
Sex (male/female)	144/46 (75.8/24.2)	69/30 (69.7/30.3)	71/31 (69.6/30.4)	68/62 (52.3/47.7)	*χ*^2^ = 20.07	*p* = 0.058
Smoking (yes/no)	132/58 (69.5/30.5)	57/4*2* (57.6/42.4)	59/43 (57.8/42.2)	74/56 (56.9/43.1)	*χ*^2^ = 7.47	*p* < 0.001
Age (years)	37.0 (19–71)	40.0** (20–69)	42.0** (19–71)	41.5** (19–82)	*H* = 12.81	*p* = 0.005
Duration of disease (years)	8 (1–31)	10 (1–31)	10 (4–25)	7 (1–30)	*H* = 8.00	*p* = 0.046
Number of episodes	4 (0–20)	4 (1–20)	5 (1–20)	4 (0–31)	*H* = 5.76	*p* = 0.124
Total PANSS_0–6_ scores (week 0)	85.5* (47–141)	83.0*^#^ (43–144)	94.0^#^ (47–141)	80.5 (47–141)	*H* = 14.43	*p* = 0.002
Total PANSS_0–6_ scores (week 8)	39.0* (15–93)	36.0* (5–89)	47.0 (15–93)	32.0* (4–92)	*H* = 14.56	*p* = 0.002
PANSS_0–6_ positive scores (week 0)	23.5^#^ (9–38)	22.0 (6–39)	25.0 (9–38)	21.0* (6–35)	*H* = 10.66	*p* = 0.014
PANSS_0–6_ positive scores (week 8)	9.0* (0–25)	8.0* (0–25)	11.5 (0–25)	6.5* (0–23)	*H* = 12.32	*p* = 0.006
PANSS_0–6_ negative scores (week 0)	20.0* (11–36)	20.0* (7–35)	23.0 (11–36)	18.5* (5–36)	*H* = 21.57	*p* < 0.001
PANSS_0–6_ negative scores (week 8)	12.0*^#^ (3–29)	11.0* (2–28)	14.0 (3–29)	9.0* (1–29)	*H* = 17.86	*p* < 0.001
PANSS_0–6_ general psychopathology scores (week 0)	41.0 (16–68)	40.0 (17–73)	44.0 (16–68)	38.5* (15–72)	*H* = 8.05	*p* = 0.045
PANSS_0–6_ general psychopathology scores (week 8)	18.0* (8–46)	17.0* (2–44)	21.0 (8–46)	15.5* (0–48)	*H* = 10.85	*p* = 0.013

Categorical data was analyzed with Chi-square test (*df* = 2) and shown as n (%). Numerical data was analyzed with Kruskal–Wallis ANOVA (*df* = 3) test and shown as median (range).

*n* number of subjects, *PANSS* positive and negative syndrome scale.

*p < 0.05 vs. clozapine; ^#^*p* < 0.05 vs. other antipsychotics; **p < 0.05 vs. olanzapine (Dunn’s test).

The frequency of responders and non-responders did not differ in patients treated with olanzapine, risperidone, clozapine or other antipsychotics, when evaluated according to the 50% reduction in the PANSS total (χ^2^ = 7.200; df = 3; p = 0.066), positive (χ^2^ = 2.121 df = 3; p = 0.548) and general psychopathology (χ^2^ = 4.931; df = 3; p = 0.177) scores. However, a significant difference in the distribution of responders and non-responders was found when comparing all four groups of patients evaluated according to the reduction in the PANSS negative scores (χ^2^ = 29.95; df = 3; p < 0.001).

The frequency of the *COMT* rs4680 (χ^2^ = 0.046; df = 1; p = 0.829) or the *COMT* rs4818 (χ^2^ = 0.523; df = 1; p = 0.469) genotypes did not deviate from HWE in patients with schizophrenia. The distribution of the *COMT* rs4680 AA, AG and GG genotypes (χ^2^ = 1.654; df = 2; p = 0.437), or the *COMT* rs4818 CC, CG and GG genotypes (χ^2^ = 0.076; df = 2; p = 0.963) did not differ significantly between male and female patients. However, we evaluated the possible association of treatment response with *COMT* rs4680 and *COMT* rs4818 genotypes or haplotypes, separately in male and female patients, and we have observed no significant associations (Supplementary Tables S1–12).

Therefore, in the further analyses, patients with schizophrenia were not subdivided according to the gender. At baseline, there were no significant differences in the frequency of the *COMT* rs4680 (χ^2^ = 1.432; df = 2; p = 0.964) or *COMT* rs4818 (χ^2^ = 5.548; df = 2; P = 0.476) genotypes between patients treated with olanzapine, risperidone, clozapine or other antipsychotics.

Table 2 demonstrates the PANSS-derived response rates in steps of 25% in patients treated for 8 weeks with adequate monotherapy^19^. The frequency of response rates differed nominally between the groups treated with different antipsychotic (p = 0.035). In all treatment groups, the highest treatment response, in 43.1–51.1% of patients, was detected in the 50–74% symptom reduction category. These results confirmed that our primary cut-off, defined a priori, as a 50% reduction in the baseline PANSS scores^19^, was a good choice to detect clinically meaningful improvement.

**Table 2:** PANSS-derived response rates in patients with schizophrenia after 8 weeks of treatment with olanzapine, risperidone, clozapine or other antipsychotics.

Antipsychotic	Total *n*	PANSS-reduction				χ^2^ test
		< 25%	25–49%	50–74%	75–100%	
Olanzapine	190	18 (9.5)	58 (30.5)	96 (50.5)	18 (9.5)	*χ*^2^ = 17.98; df = 9; *p* = 0.035
Risperidone	99	11 (11.1)	27 (27.3)	49 (49.5)	1*2* (12.1)	
Clozapine	102	7 (6.9)	47 (46.1)	44 (43.1)	4 (3.9)	
Other antipsychotics	130	17 (13.1)	31 (23.8)	67 (51.5)	15 (11.5)	

Frequencies (%) are shown in parenthesis.

*n* number of subjects, *PANSS* positive and negative syndrome scale.

After 8 weeks of antipsychotic treatment, when patients were subdivided according to the treatment response and according to the *COMT* rs4680 and rs4818 genotypes, no significant differences were found in the distribution of the *COMT* rs4680 (Table 3) or *COMT* rs4818 (Table 4) genotypes in responders and non-responders treated with olanzapine, risperidone, clozapine or other antipsychotics.

**Table 3:** The *COMT* rs4680 genotype count and frequencies in schizophrenia patients treated with olanzapine, risperidone, clozapine or other antipsychotics, subdivided into responders (R) and non-responders (NR) according to the 50% reduction in the baseline PANSS_0–6_ total and subscale scores.

*COMT* rs4680		Olanzapine *n* = 190			Risperidone *n* = 99			Clozapine *n* = 102			Other antipsychotics *n* = 130		
		AA	AG	GG	AA	AG	GG	AA	AG	GG	AA	AG	GG
Total PANSS_0–6_ score reduction at week 8	NR	17 (22.4)	35 (46.1)	24 (31.6)	13 (34.2)	15 (39.5)	10 (26.3)	13 (24.1)	26 (48.1)	15 (27.8)	13 (27.1)	21 (43.8)	14 (29.2)
	R	29 (25.4)	64 (56.1)	21 (18.4)	13 (21.3)	31 (50.8)	17 (27.9)	13 (27.1)	22 (45.8)	13 (27.5)	17 (20.7)	44 (53.7)	21 (25.6)
		*χ*^2^ = 4.40; *p* = 0.111			*χ*^2^ = 2.15; *p* = 0.341			*χ*^2^ = 0.12; *p* = 0.940			*χ*^2^ = 1.27; *p* = 0.753		
PANSS_0–6_ positive scores reduction at week 8	NR	12 (23.1)	22 (42.3)	18 (34.6)	9 (32.1)	12 (42.9)	7 (25.0)	8 (22.2)	16 (44.4)	12 (33.3)	10 (25.6)	17 (43.6)	12 (30.8)
	R	34 (34.6)	77 (55.8)	27 (19.6)	17 (23.9)	34 (47.9)	20 (28.2)	18 (27.3)	32 (48.5)	16 (24.2)	20 (22.0)	48 (52.7)	23 (25.3)
		*χ*^2^ = 4.97; *p* = 0.083			*χ*^2^ = 0.70; *p* = 0.706			*χ*^2^ = 1.02; *p* = 0.602			*χ*^2^ = 0.92; *p* = 0.630		
PANSS_0–6_ negative scores reduction at week 8	NR	31 (25.2)	57 (46.3)	35 (28.5)	17 (30.9)	23 (41.8)	15 (27.3)	22 (25.3)	41 (47.1)	24 (27.6)	16 (23.2)	33 (47.8)	20 (29.0)
	R	15 (22.4)	42 (62.7)	10 (14.9)	9 (20.5)	23 (52.3)	12 (27.3)	4 (26.7)	7 (46.7)	4 (26.7)	14 (23.0)	32 (52.5)	15 (24.6)
		*χ*^2^ = 5.72; *p* = 0.057			*χ*^2^ = 1.59; *p* = 0.451			*χ*^2^ = 0.01; *p* = 0.993			*χ*^2^ = 0.37; *p* = 0.830		
PANSS_0–6_ general psychopathology scores reduction at week 8	NR	22 (26.5)	36 (43.4)	25 (30.1)	13 (31.0)	17 (40.5)	12 (28.6)	11 (19.3)	29 (50.9)	17 (29.8)	17 (28.8)	25 (42.4)	17 (28.8)
	R	24 (22.4)	63 (58.9)	20 (18.7)	13 (22.8)	29 (50.9)	15 (26.3)	15 (33.3)	19 (42.2)	11 (24.4)	13 (18.3)	40 (56.3)	18 (25.4)
		*χ*^2^ = 5.06; *p* = 0.080			*χ*^2^ = 1.22; *p* = 0.544			*χ*^2^ = 2.61; *p* = 0.271			*χ*^2^ = 2.94; *p* = 0.230		

Frequencies (%) are shown in parenthesis.

*n* number of subjects, *NR* non-responders, *PANSS* positive and negative syndrome scale, *R* responders.

**Table 4:** The *COMT* rs4818 genotype count and frequencies in schizophrenia patients treated with olanzapine, risperidone, clozapine or other antipsychotics, subdivided into responders (R) and non-responders (NR) according to the 50% reduction in PANSS_0–6_ total and subscale scores.

*COMT* rs4818		Olanzapine *n* = 190			Risperidone *n* = 99			Clozapine *n* = 102			Other antipsychotics *n* = 130		
		CC	GC	GG	CC	GC	GG	CC	GC	GG	CC	GC	GG
Total PANSS_0–6_ scores reduction at week 8	NR	29 (38.2)	29 (38.2)	18 (23.7)	18 (47.4)	14 (36.8)	6 (15.8)	16 (29.6)	29 (53.7)	9 (16.7)	22 (45.8)	17 (35.4)	9 (18.8)
	R	36 (31.6)	62 (54.4)	16 (14.0)	22 (36.1)	30 (49.2)	9 (14.8)	15 (31.3)	25 (52.1)	8 (16.7)	34 (43.1)	36 (40.8)	12 (14.6)
		*χ *^2^ = 5.46; *p* = 0.065			*χ*^2^ = 1.56; *p* = 0.459			*χ*^2^ = 0.04; *p* = 0.983			*χ*^2^ = 0.97; *p* = 0.611		
PANSS_0–6_ positive scores reduction at week 8	NR	19 (36.5)	21 (40.4)	12 (23.1)	12 (42.9)	11 (39.3)	5 (17.9)	8 (22.2)	20 (55.6)	8 (22.2)	18 (37.5)	16	5 (62.5)
	R	46 (33.3)	70 (50.7)	22 (15.9)	28 (39.4)	33 (46.5)	10 (14.1)	23 (34.8)	34 (51.5)	9 (13.6)	38 (41.8)	37 (40.7)	16 (17.6)
		*χ*^2^ = 2.03; *p* = 0.362			*χ*^2^ = 0.48; *p* = 0.786			*χ*^2^ = 2.32; *p* = 0.313			*χ*^2^ = 0.51; *p* = 0.776		
PANSS_0–6_ negative scores reduction at week 8	NR	43 (35.0)	54 (43.9)	26 (21.1)	24 (43.6)	23 (41.8)	8 (14.5)	27 (31.0)	46 (52.9)	14 (16.1)	33 (47.8)	26 (37.7)	10 (14.5)
	R	22 (32.8)	37 (55.2)	8 (11.9)	16 (36.4)	21 (47.7)	7 (15.9)	4 (26.7)	8 (53.3)	3 (20.0)	23 (37.7)	27 (44.3)	11 (18.0)
		*χ*^2^ = 3.27; *p* = 0.195			*χ*^2^ = 0.54; *p* = 0.763			*χ*^2^ = 0.20; *p* = 0.906			*χ*^2^ = 1.37; *p* = 0.505		
PANSS_0–6_ general psychopathology scores reduction at week 8	NR	32 (38.6)	32 (38.6)	19 (22.9)	19 (45.2)	15 (35.7)	8 (19.0)	13 (22.8)	34 (59.6)	10 (17.5)	27 (45.8)	21 (35.6)	11 (18.6)
	R	33 (30.8)	59 (55.1)	15 (14.0)	21 (36.8)	29 (50.9)	7 (12.3)	18 (40.0)	20 (44.4)	7 (15.6)	29 (40.8)	32 (45.1)	10 (14.1)
		*χ*^2^ = 5.55; *p* = 0.062			*χ*^2^ = 2.40; *p* = 0.301			*χ*^2^ = 3.60; *p* = 0.165			*χ*^2^ = 1.31; *p* = 0.521		

Frequencies (%) are shown in parenthesis.

*n* number of subjects, *NR* non-responders, *PANSS* positive and negative syndrome scale, *R* responders.

To further evaluate this negative finding, we calculated the percentage of the reduction from the initial PANSS_0–6_ total and subscale scores after 8 weeks of treatment with olanzapine, risperidone, clozapine or other antipsychotics in schizophrenic patients subdivided according to the *COMT* rs4680 (Table 5) and *COMT* rs4818 (Table 6) genotypes. Nominally significant differences were detected between olanzapine-treated patients carrying *COMT* rs4680 AA, GA and GG genotypes in the total PANSS_0–6_ scores (p = 0.019; Kruskal Wallis ANOVA and Dunn’s test), and in the PANSS_0–6_ positive subscale scores (p = 0.027). However, this significance was lost due to the Bonferroni correction. Namely, carriers of the *COMT* rs4680 GA genotype had more pronounced reduction in the total PANSS_0–6_ scores compared to GG carriers (Table 5). To further evaluate this result, we additionally subdivided responders and non-responders into *COMT* rs4680 A carriers (the combined group of AA and AG genotype carriers) and GG homozygous genotype carriers. Mann–Whitney test revealed a significant difference in the PANSS_0–6_ total scores (U = 2,375.5; p < 0.006) and a trend of significance in PANSS_0–6_ positive scores (U = 2,394.5; p = 0.007), but no significant differences in the PANSS_0–6_ general psychopathology (U = 2,666.0; p = 0.043) or PANSS_0–6_ negative (U = 2,611.0; p = 0.064) scores between *COMT* rs4680 A carriers vs. GG carriers treated with olanzapine. Collectively, *COMT* rs4680 A allele carriers displayed larger percentage of the reduction in all PANSS_0–6_ scores when compared to GG carriers. Other PANSS_0–6_ scores did not differ significantly between schizophrenic patients, carriers of the *COMT* rs4680 genotypes treated with risperidone, clozapine or other antipsychotics (Table 5). Carriers of the *COMT* rs4818 genotypes had similar PANSS_0–6_ scores in all treatment’s groups (Table 6).

**Table 5:** Percentage of reduction from the initial PANSS_0–6_ total and subscale scores after 8 weeks of treatment with olanzapine, risperidone, clozapine or other antipsychotics in schizophrenia patients subdivided according to the *COMT* rs4680 genotypes.

	Olanzapine *n* = 190			Risperidone *n* = 99			Clozapine *n* = 102			Other antipsychotics *n* = 130		
	Median	Range	IR	Median	Range	IR	Median	Range	IR	Median	Range	IR
**Total PANSS**_**0–6**_** scores reduction after 8 weeks of treatment**												
*COMT* rs4680												
AA	56.0	83	33	52.4	83	32	50.1	55	23	54.3	83	40
AG	58.2	81	24	58.0	70	28	49.2	66	22	60.0	84	28
GG	49.5	71	23	63.2	76	37	45.5	53	27	54.2	79	33
Kruskal–Wallis ANOVA	*H* = 7.93; *p* = 0.019			*H* = 0.37; *p* = 0.832			*H* = 0.04; *p* = 0.981			*H* = 1.37; *p* = 0.505		
**PANSS**_**0–6**_** positive symptom scores reduction after 8 weeks of treatment**												
*COMT* rs4680												
AA	64.5	81	34	62.2	82	30	52.9	75	21	67.6	88	47
AG	63.6	79	31	61.4	86	31	57.7	89	27	64.3	83	30
GG	52.9	84	26	68.8	84	27	53.3	74	28	60.9	90	40
Kruskal–Wallis ANOVA	*H* = 7.26; *p* = 0.027			*H* = 0.18; *p* = 0.912			*H* = 0.33; *p* = 0.850			*H* = 0.54; *p* = 0.763		
**PANSS**_**0–6**_** negative symptom scores reduction after 8 weeks of treatment**												
*COMT* rs4680												
AA	37.7	80	33	37.2	89	47	28.9	73	13	37.9	88	55
AG	43.5	86	37	48.1	189	41	28.9	67	23	47.6	86	55
GG	36.4	96	29	44.0	92	42	28.2	72	22	44.4	80	47
Kruskal–Wallis ANOVA	*H* = 3.88; *p* = 0.144			*H* = 1.85; *p* = 0.398			*H* = 0.15; *p* = 0.930			*H* = 0.12; *p* = 0.944		
**PANSS**_**0–6**_** general psychopathology scores reduction after 8 weeks of treatment**												
*COMT* rs4680												
AA	51.2	95	34	48.4	86	26	51.9	58	24	44.4	83	30
AG	55.9	93	25	56.8	86	30	45.4	74	23	56.3	87	28
GG	48.3	75	17	53.6	78	39	43.9	46	25	50.0	106	34
Kruskal–Wallis ANOVA	*H* = 5.218; *p* = 0.074			*H* = 0.22; *p* = 0.897			*H* = 1.60; *p* = 0.449			*H* = 3.61; *p* = 0.164		

Values are given as median, range and interquartile range (IR).

*n* number of subjects, *PANSS* positive and negative syndrome scale.

**Table 6:** Percentage reduction from the initial PANSS_0–6_ total and subscale scores after 8 weeks of treatment with olanzapine, risperidone, clozapine or other antipsychotics in schizophrenic patients subdivided according to the *COMT* rs4818 genotypes.

	Olanzapine *n* = 190			Risperidone *n* = 99			Clozapine *n* = 102			Other antipsychotics *n* = 130		
	Median	Range	IR	Median	Range	IR	Median	Range	IR	Median	Range	IR
**Total PANSS**_**0–6**_** scores reduction after 8 weeks of treatment**												
*COMT* genotype												
CC	53.9	83	32	56.5	79	32	49.5	59	27	55.5	84	32
CG	59.0	81	23	58.1	72	22	48.3	61	23	58.4	78	30
GG	49.5	60	16	62.3	76	47	45–1	53	26	63.9	71	37
Kruskal–Wallis ANOVA	*H* = 5.32; *p* = 0.070			*H* = 0.07; *p* = 0.965			*H* = 0.01; *p* = 0.994			*H* = 1.94; *p* = 0.378		
**PANSS**_**0–6**_** positive symptom scores reduction after 8 weeks of treatment**												
*COMT* genotype												
CC	62.1	82	33	61.9	84	30	52.2	75	25	64.6	90	35
CG	66.7	88	32	65.4	81	25	59.7	79	28	63.0	86	33
GG	53.4	79	25	68.8	84	40	51.6	74	27	77.8	85	41
Kruskal–Wallis ANOVA	*H* = 4.44; *p* = 0.108			*H* = 0.03; *p* = 0.984			*H* = 1.27; *p* = 0.531			*H* = 2.25; *p* = 0.325		
**PANSS**_**0–6**_** negative symptom scores reduction after 8 weeks of treatment**												
*COMT* genotype												
CC	38.9	86	34	39.4	89	43	28.6	73	12	42.1	88	49
CG	42.3	104	37	45.5	189	49	28.6	67	24	50.0	85	47
GG	36.1	79	28	47.1	92	48	34.8	72	24	50.0	72	52
Kruskal–Wallis ANOVA	*H* = 1.93; *p* = 0.381			*H* = 0.62; *p* = 0.734			*H* = 1.56; *p* = 0.460			*H* = 1.37; *p* = 0.504		
**PANSS**_**0–6**_** general psychopathology scores reduction after 8 weeks of treatment**												
*COMT* genotype												
CC	51.2	91	30	51.0	84	29	52.3	68	23	50.0	83	31
CG	55.9	93	26	57.7	86	26	43.7	66	25	53.7	87	28
GG	48.7	70	11	48.7	78	46	44.7	46	25	47.6	106	41
Kruskal–Wallis ANOVA	*H* = 4.53; *p* = 0.104			*H* = 0.09; *p* = 0.957			*H* = 3.20; *p* = 0.202			*H* = 2.08; *p* = 0.354		

Values are given as median, range and interquartile range (IR)*n* number of subjects, *PANSS* positive and negative syndrome scale.

## Haplotype analysis

**Figure 1:**
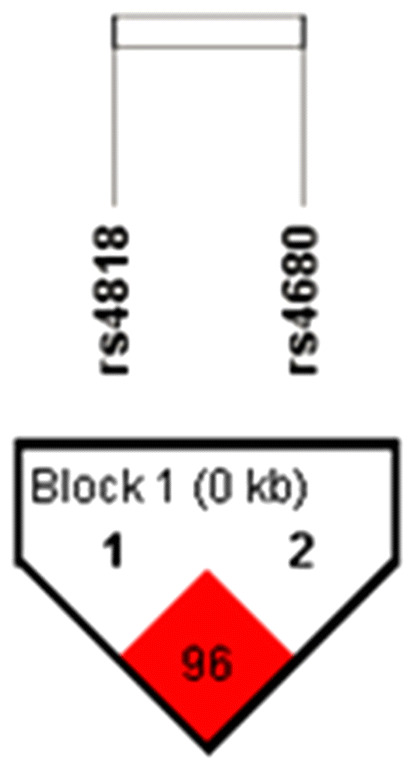
Linkage disequilibrium (LD) plot for two *COMT* SNPs in schizophrenia patients. Pairwise LD value (D’ × 100) is calculated using 4-gamete rule and represented in red square. Revealed D’ value indicates a strong link between rs4818 and rs4680 loci.

LD plot for two analyzed *COMT* SNPs was determined with Haploview software v. 4.2 and shown in Fig. 1.

Since *COMT* rs4818 and rs4680 loci were highly linked (D′ = 0.96; LOD > 2), the frequencies of four possible haplotypes were calculated (Fig. 1). Frequencies of various *COMT* (rs4818–rs4680) haplotypes in patients with schizophrenia, estimated with Haploview v 4.2., were C-A (0.488); G-G (0.369); C-G (0.124) and G-A (0.008, less than 1%). Rare haplotype (< 1%) was excluded from the analysis.

To further evaluate this significant association between the *COMT* rs4680 A allele and better response to olanzapine treatment, we compared the frequency of the most common *COMT* C-A haplotype with other haplotype carriers (Table 7), subdivided into responders and non-responders. Nominally significant differences were found in the frequency of the *COMT* C-A haplotype carriers and other haplotype carriers between responders and non-responders to olanzapine (Table 7), defined according to the reduction in the PANSS_0–6_ total scores (p = 0.036), PANSS_0–6_ positive subscale scores (p = 0.021), and PANSS_0–6_ negative subscale scores (p = 0.050). These findings did not remain significant after correction for multiple testing. In the case of the PANSS_0–6_ general psychopathology scores, there was no difference (p = 0.064) in the frequency of *COMT* C-A haplotype carriers vs. other haplotype carriers in patients who responded well to the therapy with olanzapine and non-responders. Similar distribution of the *COMT* rs4680–rs4818 haplotypes was found in responders and non-responders to risperidone, clozapine and other antipsychotic medication (Table 7).

**Table 7:** Haplotype frequencies of *COMT* rs4680 and rs4818 polymorphisms in schizophrenia patients treated with olanzapine, risperidone, clozapine or other antipsychotics, subdivided into responders (R) and non-responders (NR) according to the 50% reduction in PANSS_0–6_ total and subscale scores.

*COMT* rs4680–rs4818		Olanzapine *n* = 190		Risperidone *n* = 99		Clozapine *n* = 102		Other antipsychotics *n* = 130	
C-A haplotype carriers		C-A carriers	Non-carriers	C-A carriers	Non-carriers	C-A carriers	Non-carriers	C-A carriers	Non-carriers
Total PANSS_0–6_ scores reduction at week 8	NR	51 (61.7)	25 (32.9)	28 (73.7)	10 (26.3)	39 (72.2)	15 (27.8)	32 (66.7)	16 (33.3)
	R	92 (80.7)	22 (19.3)	44 (72.1)	17 (27.9)	35 (72.9)	13 (27.1)	60 (73.2)	22 (26.8)
		*χ*^2^ = 4.53; *p* = 0.033		*χ*^2^ = 0.03; *p* = 0.866		*χ*^2^ = 0.01; *p* = 0.937		*χ*^2^ = 0.62; *p* = 0.431	
PANSS_0–6_ positive scores reduction at week 8	NR	33 (63.5)	19 (36.5)	21 (75.0)	7 (25.0)	24 (66.7)	12 (33.3)	26 (66.7)	13 (33.3)
	R	110 (79.7)	28 (20.3)	51 (71.8)	20 (28.2)	50 (75.8)	16 (24.2)	66 (72.5)	25 (27.5)
		χ^2^ = 5.36; p = 0.021		*χ*^2^ = 0.11; *p* = 0.750		*χ*^2^ = 0.97; *p* = 0.326		*χ*^2^ = 0.45; *p* = 0.501	
PANSS_0–6_ negative scores reduction at week 8	NR	87 (70.7)	36 (29.3)	40 (72.7)	15 (27.3)	63 (72.4)	24 (27.6)	47 (68.1)	22 (31.9)
	R	56 (83.6)	11 (16.4)	32 (72.7)	12 (27.3)	11 (73.3)	4 (26.7)	45 (73.8)	16 (26.2)
		*χ*^2^ = 3.85; *p* = 0.050		*χ*^2^ = 0.00; *p* = 1.000		*χ*^2^ = 0.01; *p* = 0.941		*χ*^2^ = 0.50; *p* = 0.479	
PANSS_0–6_ general psychopathology scores reduction at week 8	NR	57 (68.7)	26 (31.3)	30 (71.4)	12 (28.6)	40 (70.2)	17 (29.8)	40 (67.8)	19 (32.2)
	R	86 (80.4)	21 (19.6)	42 (73.7)	15 (26.3)	34 (75.6)	11 (24.4)	52 (73.2)	19 (26.8)
		*χ*^2^ = 3.44; *p* = 0.064		*χ*^2^ = 0.06; *p* = 0.803		*χ*^2^ = 0.37; *p* = 0.545		*χ*^2^ = 0.46; *p* = 0.497	

Frequencies (%) are shown in parenthesis.

*n* number of subjects, *NR* non-responders, *PANSS* positive and negative syndrome scale, *R* responders.

Results presented in Table 8 revealed significantly greater reduction of total PANSS_0–6_ scores (Mann Whitney test; p < 0.006) and a trend towards larger reduction of PANSS_0–6_ positive subscale scores (p = 0.007) in *COMT* C-A haplotype carriers in olanzapine-treated patients. Slight reduction was detected in the PANSS_0–6_ general psychopathology scores (p = 0.037), when comparing *COMT* C-A haplotype carriers to the carriers of other haplotypes in patients treated with olanzapine. Other results were not significant, showing that reduction in the PANSS_0–6_ total and subscale scores did not differ between the carriers of the *COMT* C-A haplotype compared to other haplotype carriers in patients treated with risperidone, clozapine or other antipsychotics (Table 8).

**Table 8:** Percentage reduction from the initial PANSS_0–6_ total and subscale scores after 8 weeks of treatment with olanzapine, risperidone, clozapine or other antipsychotics in schizophrenic patients subdivided according to the *COMT* rs4680–rs4818 haplotypes into C-A haplotype carriers and carriers of the other haplotypes (non-carriers).

	Olanzapine *n* = 190			Risperidone *n* = 99			Clozapine *n* = 102			Other antipsychotics *n* = 130		
	Median	Range	IR	Median	Range	IR	Median	Range	IR	Median	Range	IR
**Total PANSS**_**0–6**_** scores reduction after 8 weeks of treatment**												
*COMT* rs4680–rs4818												
C-A carriers	57.7	86	26	57.3	83	30	49.5	66	23	57.9	85	32
Non-carriers	49.5	71	23	63.2	76	37	45.5	53	27	53.6	79	34
Mann–Whitney U test	*U* = 2,463.5; *p* = 0.006			*U* = 927.5; *p* = 0.727			*U* = 1,016.5; *p* = 0.884			*U* = 1701.0; *p* = 0.810		
**PANSS**_**0–6**_** positive symptom scores reduction after 8 weeks of treatment**												
*COMT* rs4680–rs4818												
C-A carriers	64.0	81	31	61.4	86	30	54.6	90	27	65.9	86	33
Non-carriers	52.9	84	26	68.8	84	27	53.3	74	28	58.3	90	40
Mann–Whitney U test	*U* = 2,474.0; *p* = 0.007			*U* = 943.0; *p* = 0.820			*U* = 984.5; *p* = 0.699			*U* = 1736.5. *p* = 0.953		
**PANSS**_**0–6**_** negative symptom scores reduction after 8 weeks of treatment**												
*COMT* rs4680–rs4818												
C-A carriers	42.9	86	37	43.35	198	48	28.9	73	19	47.4	88	56
Non-carriers	36.4	96	31	44.0	92	42	28.2	72	22	44.2	80	46
Mann–Whitney U test	*U* = 2,747.0; *p* = 0.061			*U* = 900.0; *p* = 0.571			*U* = 988.0; *p* = 0.719			*U* = 1714.0; *p* = 0.862		
**PANSS**_**0–6**_** general psychopathology scores reduction after 8 weeks of treatment**												
*COMT* rs4680–rs4818												
C-A carriers	55.0	95	25	54.2	95	28	47.3	74	21	53.8	92	27
Non-carriers	48.3	75	19	53.6	78	39	43.9	46	25	49.5	106	35
Mann–Whitney U test	*U* = 2,679.0; *p* = 0.037			*U* = 950.0; *p* = 0.863			*U* = 1,014.5; *p* = 0.872			*U* = 1621.0; *p* = 0.516		

Values are given as median, range and interquartile range (IR).

*n* number of subjects, *PANSS* positive and negative syndrome scale.

## Discussion

This longitudinal study detected a significant association of *COMT* rs4680 genotype and *COMT* rs4680–rs4818 haplotype with the much-improved therapeutic response to 8-weeks monotherapy with olanzapine in Caucasian patients with schizophrenia. Our results revealed that olanzapine treated patients, carriers of the *COMT* rs4680 A allele, or carries of the *COMT* rs4680–rs4818 C-A haplotype, had greater reduction in the PANSS_0–6_ total scores. Similar, but statistically non-significant trend, was observed in the *COMT* rs4680 GA homozygous genotype carriers. A trend towards more significant reduction in the PANSS_0–6_ positive subscale scores was also detected for the *COMT* rs4680 A allele carriers and the *COMT* rs4680–rs4818 C-A haplotype carriers, when compared to the carriers of the *COMT* rs4680 GG genotype or the carriers of other *COMT* rs4680–rs4818 haplotypes. Treatment response was determined using a priori cut-off, based on criteria suggested by Leucht et al.^19^ and based on our previous studies and clinical experience. However, treatment response was also determined by the observed percentage reduction from the initial PANSS_0–6_ total and subscale scores. This was done in order to avoid potential error by determining the treatment response only by a specific cut-off. From our results it is visible that both approaches yielded similar results and trends. This was observed in the case of *COMT* rs4680 and treatment response to olanzapine (Tables 3,5), but also in the case of haplotype analysis (Tables 7,8). However, most of these nominally significant differences were lost after correcting for multiple testing, but the detected trend in a treatment response was the same when we used either a priori cut-off or the percentage reduction from the initial PANSS_0–6_ total and subscale scores.

The association between *COMT* rs4680 A allele and better response to treatment with olanzapine (greater reduction in the PANSS_0–6_ total and a similar trend in PANSS_0–6_ positive subscale scores), detected in our study, agrees with findings showing that homozygous *COMT* rs4680 AA genotype carriers with schizophrenia had faster therapeutic response to olanzapine^20, 21^. Two studies with small sample sizes, evaluating olanzapine monotherapy, reported that the *COMT* rs4680 AA carriers more effectively reduced PANSS symptoms compared to G carriers^22^. Patients with schizophrenia, carriers of the *COMT* rs4680 AA genotype had faster and better response to atypical antipsychotics than G allele carriers^11, 22–27^. Similar results to ours were reported for the Japanese patients with schizophrenia, carriers of the *COMT* rs4680 AA genotype, treated with aripiprazole for 6 weeks, who showed greater improvement in the PANSS total and general psychopathology scores^28^. In line with our data, the recent-meta analysis demonstrated that in patients treated with atypical antipsychotics, the *COMT* rs4680 AA carriers were significantly more likely to respond well to therapy compared to G allele carriers^1^. Although there are differences in the treatment response definition between our (50% reduction) and the cited study (30% reduction), the results are similar.

On the other hand, in 107 Italian patients with schizophrenia, treated with clozapine, carriers of the *COMT* rs4680 GG genotype showed a greater improvement in the PANSS negative subscale score (but not in other PANSS subscales scores), compared to both GA or AA genotype carriers^29^. In our study, including 102 patients treated with clozapine, no significant association was detected between the *COMT* rs4680 genotype and treatment response or improvement in negative symptoms after clozapine monotherapy. These differences might be explained by the use of different criteria and different design, since we subdivided all our patients according to the treatment response into responders and non-responders, and into carriers of the *COMT* rs4680 genotypes, while the other study evaluated treatment response in resistant patients with the regression analysis, using *COMT* and *HTR1A* genotypes as predictors^29^. An additive effect of *COMT* and *HTR1A* genotypes on the improvement in the PANSS negative symptoms subscale score was suggested, and a better reduction in negative symptoms, after clozapine treatment, was found in patients who were carriers of both *COMT* rs4680 GG and *HTR1A* GG genotypes^29^.

The type of antipsychotic medication might be a possible predictor of the treatment response^1^. On the other hand, our results, showing a trend towards beneficial therapeutic effect of olanzapine in carriers of the *COMT* rs4680 GA genotype, are in line with the results of the previous study that revealed a trend, a 3 times greater prevalence of the heterozygous *COMT* rs4680 GA genotype, compared to AA or GG genotypes, in patients who achieved symptomatic remission^7^. Our results do not agree with the lack of association between *COMT* rs4680 and clinical response to antipsychotics, including olanzapine^15, 16^. Opposed to our data, higher frequency of the *COMT* rs4680 G allele was found in the responders compared to ultra-resistant patients of the Mexican origin^30^. The discrepancies might be due to the ethnic origin of the patients, as well as to duration and definition of the response, remission and ultra-resistance.

In a haplotype analysis we have detected a significant association of the *COMT* C-A haplotype (rs4818–rs4680) and treatment response to olanzapine, but not to risperidone, clozapine or other antipsychotics. This *COMT* haplotype (C-A) was reported to be significantly related to a good response to risperidone^11^. In contrast to these results, in our study, including 99 patients treated with risperidone, the *COMT* C-A haplotype was not significantly associated with treatment response. The differences might be due to different ethnicity and treatment response definition, since we included Caucasian patients, with treatment response as 50% reduction of the PANSS baseline scores, while the other study included subjects of South Indian origin and defined treatment response as the reduction to the scores of two or less on the CGI Global Improvement scale^11^. In our previous study on the treatment resistant schizophrenia^7^, the G alleles of the *COMT* rs4680 and rs4818, as well as the high activity *COMT* G-G/G-G haplotype, had lower risk to become treatment resistant only in female but not in male patients with schizophrenia. In contrast to these data^7^, in the present study the genotype distribution for the *COMT* rs4680 and rs4818 did not differ according to gender. However, when male and female subjects were evaluated separately, no significant association was detected between the *COMT* rs4680 and rs4818 genotypes and haplotypes and treatment response to olanzapine, risperidone, clozapine or other antipsychotics (Supplementary Tables S1–S12). In addition, in contrast to our previous study^7^, in the present study we evaluated treatment response and not treatment resistance. The differences between these studies are in the design, since present study was longitudinal while our previous study was cross-sectional, and, unlike the current investigation, previous study did not exclude patients who received ECT, as well as different antipsychotic combinations^7^. Large heterogeneity across studies, adherence to treatment, population stratification^6^, as well as influence of other functional variants in the *COMT* gene^7, 31, 32^, interactions with other gene polymorphisms, such as *DRD4* (120–bp duplication)^33^, might explain some of the inconsistent findings. In addition, recent studies^34, 35^ discussed the disadvantages of the candidate gene association studies compared to genome-wide association studies (GWAS), pointing to the problems of high false discovery rate, low replication rates and insufficient knowledge to correctly identify possible candidate genes. However, in the case of *COMT* polymorphisms, meta-analyses demonstrated the consistent effects on the treatment outcome in schizophrenia^1, 6^, but with small effect sizes and limited predictive power. Due to these inconsistent data on the association between *COMT* variants and treatment response to different antipsychotics in schizophrenia, we evaluated genotype- and haplotype-based association of the *COMT* rs4680 and rs4818 polymorphisms with the much better treatment response in schizophrenia.

The limitations of the study should be acknowledged. Although the sample size was respectable (N = 521), when stratified according to the individual antipsychotic medication, the study included 190 olanzapine-, 99 risperidone-, 102 clozapine-treated patients and 130 patients treated with other antipsychotics, which is lower than the calculated required sample size for each medication group. Moreover, the association between treatment response and only two *COMT* polymorphisms (rs4680 and 4818) was analyzed, while not taking into account that other *COMT* polymorphisms or polymorphisms of other dopaminergic genes might affect treatment response in a polygenic multi-factorial disorder, such as schizophrenia. On the other hand, *COMT* rs4680 was recently^3^ confirmed to be a functional polymorphism, since it significantly affects abundance, stability, and activity of the COMT enzyme^36^. There is also evidence of a large inter-individual variation in the pharmacokinetics of olanzapine, leading to multiple differences in drug exposure between subjects at a given dose, which might explain the concentration-dependent therapeutic failures between studies. However, in our study we were unable to determine plasma concentration of olanzapine in patients with schizophrenia. Non-replication of the pharmacogenetic data is common, due to different study designs, small sample sizes, lack of statistical power, ethnic and racial differences, small effects of the most individual genes, variety of environmental and clinical confounders, differences in definition of response, remission or resistance, and lack of evaluation of the possible effects of other gene polymorphisms^4, 6, 36^.

Strengths of the present study are in both genotype and haplotype analyses, olanzapine, risperidone, and clozapine monotherapy, inclusion of ethnically homogenous Caucasian patients with schizophrenia, usage of a priori cut-off point of 50% reduction from the baseline PANSS total and subscale scores^19^ and the percentage reduction from the initial PANSS_0–6_ scores for the treatment response, corrected p-value, evaluation of the possible sex differences, and the longitudinal study design (including 8 weeks follow up). Unlike previous studies, which investigated treatment response to different antipsychotics, the present investigation focused on the substantial response to olanzapine, risperidone or clozapine monotherapy. Therefore, our results confirmed a significant association of the *COMT* rs4680 A allele, and the *COMT* rs4680–rs4818 C-A haplotype, with a good therapeutic response to olanzapine. These data offer pharmacogenetic information for clinicians, with a predictive value to choose responsive patients for the treatment with olanzapine, in a quest to find reliable genetic markers of treatment outcome in schizophrenia.

## Methods

### Participants

The clinical characteristics of the study sample were described in detail in our previous study^37^. Diagnosis of schizophrenia was conducted using a structured clinical interview^38^ based on the DSM-IV criteria. The present study included 521 patients (67.6% males) who were 40.3 ± 12.0 years old (range 19–82), and part of them (N = 87) were included in our previous longitudinal 6 months study evaluating remission and not therapeutic response^9^. Before the study, patients with schizophrenia were treated with different antipsychotics: olanzapine (5–20 mg/day), clozapine (100–800 mg/day), risperidone (2–6 mg/day), fluphenazine (5–15 mg/day), haloperidol (4–15 mg/day), promazine (50–300 mg/day), quetiapine (300–800 mg/day), ziprasidone (80–160 mg/day), amisulpride (200–600 mg/day), sulpiride (200–800 mg/day), sertindole (12–16 mg/day), zuclopenthixol (20–40 mg/day), alone or in combination with benzodiazepines, i.e. diazepam (5–30 mg/day). At some point, some patients were also previously treated with long-acting antipsychotics (LAI): olanzapine 210–405 mg monthly, risperidone LAI 25–50 mg monthly, fluphenazine LAI 25–50 mg monthly, haloperidol LAI 50–100 mg monthly, zuclopenthixol depot (150–300 mg monthly). All depot preparations were discontinued at least a month prior to inclusion in the study, whilst majority of such patients stopped receiving LAIs several months before entering this trial. After inclusion in the study, patients were subdivided, under the discretion of their psychiatrist, into groups treated with olanzapine (10–20 mg/day); N = 190 (36.5%), risperidone (3–6 mg/day); N = 99 (19.0%), or clozapine (100–500 mg/day); N = 102 (19.6%) monotherapy for 8 weeks. The fourth group, due to the small sample sizes was merged into one group designated as “other antipsychotics” (N = 130; 25%), consisted of patients receiving monotherapy with haloperidol (3–15 mg/day) or fluphenazine (4–25 mg/day) or quetiapine (50–800 mg/day). All patients received monotherapy for 8 weeks. During the study no concomitant medication was allowed except benzodiazepines when needed. Patients were excluded from the study in the case of exacerbation of the illness, or the need to add additional antipsychotic or switching to another antipsychotic medication. Patients were evaluated with structured interview for the Positive and Negative Syndrome Scale (PANSS) including the PANSS positive, PANSS negative and PANSS general psychopathology subscales^39^. Patients were included if they were in- and out-patients diagnosed with schizophrenia for at least 5 years; treated with monotherapy with the above listed antipsychotic drugs, with added benzodiazepines when needed; patients who finished 8 weeks of treatment; who were ≥ 18 years old and who signed informed consent. Exclusion criteria were the use of antidepressants and polytherapy with antipsychotics, intellectual disabilities, patients with first-episode psychosis and patients with mild symptoms at baseline (baseline PANSS_1–7_ score ≤ 58), substance abuse and dependence in the previous 3 months, patients with any comorbid severe somatic or neurological disorder and patients who had no available detailed medical records with complete psychiatric medication history. After inclusion, all patients underwent complete diagnostic evaluation. Evaluation of the treatment response was conducted using the PANSS total and subscale scores at baseline (during the first few days of admission) and after 8 weeks of treatment. All raters were blind to genotyping data. Interrater reliability was 97%. Patients were sampled from the two centers (University Hospital Center Zagreb and Clinics for Psychiatry Vrapce, Zagreb) and were all ethnically homogenous unrelated Caucasians of European ancestry (of Croatian origin). Before the study, treatment response was defined as ≥ 50% reduction from the baseline PANSS total and subscale scores^19^. The scoring system of the PANSS was corrected from values of 1–7 to values of 0–6 as suggested^19^. At baseline, median corrected PANSS_0–6_ total score was 86.0 (range 43–144), while after 8 weeks of treatment, it was 39.0 (range 4–116).

The local Ethics committees from University Hospital Center Zagreb and Clinics for Psychiatry Vrapce, Zagreb, approved the study. After explaining the aims and procedures of the study, all participants signed the informed consent. The study did not include the patients that needed legally authorized representative for signing the informed consent. All human studies were performed with the full cooperation and understanding of the participants. The study was performed in accordance with the ethical standards laid down in the 1964 Declaration of Helsinki.

### Blood collection and genotyping

Blood samples were collected during routine laboratory visits. Genomic DNA was extracted from peripheral blood using a salting out method^40^. The genotyping of the *COMT* rs4680 (assay ID: C_25746809_50) and *COMT* rs4818 (assay ID: C_2538750_10) was performed according to the procedures described by Applied Biosystems. Researchers were blind to clinical data. We used the primers and probes from the TaqMan R Drug Metabolism Genotyping Assays (Applied Biosystems, Foster City, CA, United States), and detection was performed on ABI Prism 7300 Real time PCR System apparatus (Applied Biosystems, Foster City, CA, United States). The reaction volume of 10 mL contained 30–100 ng of DNA. As a quality control, we randomly selected up to 10% of samples and genotyped them again.

### Statistical evaluation

Data were analyzed using Sigma Stat 3.5 (Jandel Scientific Corp. San Rafael, CA, USA) and Microsoft Excel. Data distribution normality was determined with the Kolmogorov–Smirnov normality test. Due to the lack of a normal distribution, Kruskal–Wallis analysis of variance (ANOVA) and Dunn post hoc were used to assess differences in age, PANSS total, positive, negative, and general psychopathology scores between different groups of patients. The Hardy–Weinberg equilibrium (HWE), as well as genotype and haplotype distributions, were determined using χ^2^-test^41^. Haploview software v. 4.2^42^ was used to determine LD pairwise values for *COMT* rs4818 and rs4680 polymorphisms. Loci were considered to be in linkage disequilibrium if D’ coefficient was > 0.80. Haplotype was estimated for every patient by PLINK v. 1.07 software using the expectation–maximization algorithm^43^.

For individual SNP analysis the p-value (0.05/8 = 0.00625) was corrected because two SNPs were analyzed and treatment response was tested in 4 medication groups. The results were considered significant if *p* < 0.00625.

G ∗ Power 3 Software^44^ was used to conduct the power analysis. For a χ^2^-test [with α = 0.006; with expected medium effect size = 0.3; power (1 − β) = 0.800] the required sample size was N = 188 for df = 3; N = 169 for df = 2; or N = 144 for df = 1. For ANOVA [with α = 0.006; with expected medium effect size = 0.25; and power (1 − β) = 0.800] the required sample size was N = 276 for 4 compared groups and N = 249 for 3 compared group. For t-test [with α = 0.006; with expected medium effect size = 0.5; power (1 − β) = 0.800] the required sample size was N = 184.

## Supplementary information

Supplementary file1
