# Supplementary material for: Catechol-O-methyltransferase rs4680 and rs4818 haplotype association with treatment response to olanzapine in patients with schizophrenia
Source: Sci Rep. 2020 Jun 22;10:10049. doi: 10.1038/s41598-020-67351-5 (PMC7308339; doi:10.1038/s41598-020-67351-5)
Supplement: Supplementary file 1 — Supplementary file1 [file 41598_2020_67351_MOESM1_ESM.docx]

**Specific genotype and haplotype variants of catechol-O-methyltransferase are associated with treatment response to olanzapine in patients with schizophrenia**

Matea Nikolac Perkovic^1^, Marina Sagud^2,3^, Maja Zivkovic^2^, Suzana Uzun^4,5^, Gordana Nedic Erjavec^1^, Oliver Kozumplik^4,5^, Dubravka Svob Strac^1^, Ninoslav Mimica^3,4^, Alma Mihaljevic Peles^2,3^ and Nela Pivac^1^*

^1^Division of Molecular Medicine, Rudjer Boskovic Institute, Bijenicka 54, 10000 Zagreb, Croatia

^2^University Hospital Center Zagreb, Kispaticeva 12, 10000 Zagreb, Croatia

^3^School of Medicine, University of Zagreb, Salata 3, 10000 Zagreb, Croatia

^4^Department of General Psychiatry, Clinics for Psychiatry Vrapce, Bolnicka cesta 32, 10000 Zagreb, Croatia

^5^Faculty of Medicine, Josip Juraj Strossmayer University, Josipa Huttlera 4, 31000 Osijek, Croatia

Supplementary Table S1. The *COMT* rs4680 genotype count and frequencies in male schizophrenia patients treated with olanzapine, risperidone, clozapine or other antipsychotics, subdivided into responders (R) and non-responders (NR) according to the 50% reduction in the baseline PANSS_0-6_ total and subscale scores.

|  |  | Olanzapine  *n* = 144 | | |  | Risperidone  *n* = 69 | | |  | Clozapine  *n* = 71 | | |  | Other antipsychotics  *n* = 68 | | |
| --- | --- | --- | --- | --- | --- | --- | --- | --- | --- | --- | --- | --- | --- | --- | --- | --- |
| *COMT* rs4680 |  | AA | AG | GG |  | AA | AG | GG |  | AA | AG | GG |  | AA | AG | GG |
| Total PANSS_0-6_ score reduction at week 8 | NR | 14 (23.7) | 27 (45.8) | 18 (30.5) |  | 12 (40.0) | 9  (30.0) | 9 (30.0) |  | 9 (20.0) | 24 (53.3) | 12 (26.7) |  | 10 (27.8) | 16 (44.4) | 10 (27.8) |
|  | R | 21 (24.7) | 47 (55.3) | 17 (20.0) |  | 11 (28.2) | 20 (51.3) | 8 (42.0) |  | 7 (26.9) | 14 (53.8) | 5 (19.2) |  | 7 (21.9) | 18 (56.3) | 7 (21.9) |
|  |  | *χ ^2^* = 2.21; *p* =0.331 | | |  | *χ^2^* = 3.15; *p* = 0.207 | | |  | *χ^2^* = 0.73; *p* = 0.694 | | |  | *χ^2^* = 0.94; *p* = 0.624 | | |
| PANSS_0-6_ positive scores reduction at week 8 | NR | 10 (25.6) | 16 (41.0) | 13 (33.3) |  | 8 (36.4) | 7 (31.8) | 7 (31.8) |  | 4 (13.3) | 15 (50.0) | 11 (36.7) |  | 7  (25.9) | 12 (44.4) | 8 (29.6) |
|  | R | 25  (23.8) | 58 (55.2) | 22 (21.0) |  | 15 (31.9) | 22 (46.8) | 10 (21.3) |  | 12 (29.3) | 23 (56.1) | 6 (14.6) |  | 10 (24.4) | 22 (53.7) | 9 (22.0) |
|  |  | *χ^2^* = 2.95; *p* = 0.229 | | |  | *χ^2^* = 1.57; *p* = 0.457 | | |  | *χ^2^* = 5.59; *p* = 0.061 | | |  | *χ^2^* = 0.68; *p* = 0.713 | | |
| PANSS_0-6_ negative scores reduction at week 8 | NR | 24 (25.5) | 44  (46.8) | 26 (27.7) |  | 16 (35.6) | 16 (35.6) | 13 (28.9) |  | 16 (24.2) | 33 (50.0) | 17 (25.8) |  | 12 (25.0) | 23 (47.9) | 13 (27.1) |
|  | R | 11 (22.0) | 30 (60.0) | 9 (18.0) |  | 7 (29.2) | 13 (54.2) | 4 (16.7) |  | 0 (0.0) | 5 (100.0) | 0 (0.0) |  | 5 (25.0) | 11 (55.0) | 4 (20.0) |
|  |  | *χ^2^* = 2.53; *p* = 0.283 | | |  | *χ^2^* = 2.43; *p* = 0.297 | | |  | *χ^2^* = 4.67; *p* = 0.097 | | |  | *χ^2^* = 0.43; *p* = 0.809 | | |
| PANSS_0-6_ general psychopathology scores reduction at week 8 | NR | 17 (30.4) | 23 (41.1) | 16 (28.6) |  | 12 (40.0) | 8  (26.7) | 10 (33.3) |  | 7 (16.7) | 24 (57.1) | 11 (26.2) |  | 10 (28.6) | 15 (42.9) | 10 (28.6) |
|  | R | 18 (20.5) | 51 (58.0) | 19 (21.6) |  | 11 (28.2) | 21 (53.8) | 7 (17.9) |  | 9 (31.0) | 14 (48.3) | 6 (20.7) |  | 7 (21.2) | 19 (57.6) | 7 (21.2) |
|  |  | *χ^2^* = 9.97; *p* = 0.138 | | |  | *χ^2^* = 5.32; *p* = 0.070 | | |  | *χ^2^* = 2.04; *p* = 0.361 | | |  | *χ^2^* = 1.47; *p* = 0.479 | | |

Frequencies (%) are shown in parenthesis; *n* = number of subjects; NR = non-responders; PANSS = Positive and Negative Syndrome Scale; R = responders.

Supplementary Table S2. The *COMT* rs4818 genotype count and frequencies in male schizophrenia patients treated with olanzapine, risperidone, clozapine or other antipsychotics, subdivided into responders (R) and non-responders (NR) according to the 50% reduction in PANSS_0-6_ total and subscale scores.

|  |  | Olanzapine  *n* = 144 | | |  | Risperidone  *n* = 69 | | |  | Clozapine  *n* = 71 | | |  | Other antipsychotics  *n* = 68 | | |
| --- | --- | --- | --- | --- | --- | --- | --- | --- | --- | --- | --- | --- | --- | --- | --- | --- |
| *COMT* rs4818 |  | CC | GC | GG |  | CC | GC | GG |  | CC | GC | GG |  | CC | GC | GG |
| Total PANSS_0-6_ scores reduction at week 8 | NR | 24 (40.7) | 21 (35.6) | 14 (23.7) |  | 16 (53.3) | 8  (26.7) | 6 (20.0) |  | 15 (33.3) | 22 (61.1) | 8 (17.8) |  | 16 (44.4) | 14 (38.9) | 6 (16.7) |
|  | R | 23 (27.1) | 50 (58.8) | 12 (14.1) |  | 15 (38.5) | 20 (51.3) | 4 (10.3) |  | 8 (30.8) | 14 (53.8) | 4 (15.4) |  | 14 (43.8) | 14 (43.8) | 4 (12.5) |
|  |  | *χ ^2^* = 7.57; *p* =0.023 | | |  | *χ^2^* = 4.48; *p* = 0.107 | | |  | *χ^2^* = 0.17; *p* = 0.919 | | |  | *χ^2^* = 0.30; *p* = 0.861 | | |
| PANSS_0-6_ positive scores reduction at week 8 | NR | 16 (41.0) | 13 (33.3) | 10 (25.6) |  | 11 (50.0) | 6 (27.3) | 5 (22.7) |  | 7 (23.3) | 16 (53.3) | 7 (23.3) |  | 12  (44.4) | 11  (40.7) | 4  (14.8) |
|  | R | 31 (29.5) | 58 (55.2) | 16 (15.2) |  | 20 (42.6) | 22 (46.8) | 5 (10.6) |  | 16 (39.0) | 20 (48.8) | 5 (12.2) |  | 18 (43.9) | 17 (41.5) | 6 (14.6) |
|  |  | *χ^2^* = 5.63; *p* = 0.060 | | |  | *χ^2^* = 3.11; *p* = 0.212 | | |  | *χ^2^* = 2.66; *p* = 0.265 | | |  | *χ^2^* = 0.00; *p* = 0.998 | | |
| PANSS_0-6_ negative scores reduction at week 8 | NR | 32 (34.0) | 42 (44.7) | 20 (21.3) |  | 21 (46.7) | 16 (35.6) | 8 (17.8) |  | 23 (34.8) | 31 (47.0) | 12 (18.2) |  | 24 (50.0) | 17 (35.4) | 7 (14.6) |
|  | R | 15 (30.0) | 29 (58.0) | 6 (12.0) |  | 10 (41.7) | 12 (50.0) | 2 (8.3) |  | 0  (0.0) | 5 (100.0) | 0 (0.0) |  | 6 (30.0) | 11 (55.0) | 3 (15.0) |
|  |  | *χ^2^* = 2.89; *p* = 0.235 | | |  | *χ^2^* = 1.86; *p* = 0.396 | | |  | *χ^2^* = 5.23; *p* = 0.073 | | |  | *χ^2^* = 2.60; *p* = 0.273 | | |
| PANSS_0-6_ general psychopathology scores reduction at week 8 | NR | 25  (44.6) | 19 (33.9) | 12 (21.4) |  | 16 (53.3) | 7 (23.3) | 7 (23.3) |  | 12 (28.6) | 22 (52.4) | 8 (19.0) |  | 15  (42.9) | 14 (40.0) | 6 (17.1) |
|  | R | 22 (25.0) | 52 (59.1) | 14 (15.9) |  | 15 (38.5) | 21 (53.8) | 3 (7.7) |  | 11 (37.9) | 14 (48.3) | 4 (13.8) |  | 15 (45.5) | 14 (42.4) | 4 (12.1) |
|  |  | *χ^2^* = 9.02; *p* = 0.011 | | |  | *χ^2^* = 7.59; *p* = 0.023 | | |  | *χ^2^* = 0.80; *p* = 0.670 | | |  | *χ^2^* = 0.34; *p* = 0.843 | | |

Frequencies (%) are shown in parenthesis; *n* = number of subjects; NR = non-responders; PANSS = Positive and Negative Syndrome Scale; R = responders.

Supplementary Table S3. Percentage of reduction from the initial PANSS_0-6_ total and subscale scores after 8 weeks of treatment with olanzapine, risperidone, clozapine or other antipsychotics in male schizophrenia patients subdivided according to the *COMT* rs4680 genotypes.

|  | | Olanzapine  *n* = 144 | | |  | Risperidone  *n* = 69 | | |  | Clozapine  *n* = 71 | | |  | Other antipsychotics  *n* = 68 | | |
| --- | --- | --- | --- | --- | --- | --- | --- | --- | --- | --- | --- | --- | --- | --- | --- | --- |
|  |  | Median | Range | IR |  | Median | Range | IR |  | Median | Range | IR |  | Median | Range | IR |
| Total PANSS_0-6_ scores reduction after 8 weeks of treatment | | | | | | | | | | | | | | | | |
| *COMT* rs4680 | |  |  |  |  |  |  |  |  |  |  |  |  |  |  |  |
|  | AA | 55.4 | 83 | 35 |  | 48.6 | 79 | 30 |  | 49.5 | 42 | 13 |  | 44.0 | 56 | 44 |
|  | AG | 57.4 | 81 | 25 |  | 57.7 | 70 | 28 |  | 46.7 | 66 | 19 |  | 50.8 | 84 | 30 |
|  | GG | 49.5 | 71 | 25 |  | 49.4 | 72 | 40 |  | 41.7 | 46 | 22 |  | 45.1 | 79 | 40 |
|  | Kruskal-Wallis ANOVA | *H* = 5.04; *p* = 0.081 | | |  | *H* = 1.07; *p* = 0.585 | | |  | *H* = 1.17; *p* = 0.557 | | |  | *H* = 0.06; *p* = 0.970 | | |
| PANSS_0-6_ positive symptom scores reduction after 8 weeks of treatment | | | | | | | | | | | | | | | | |
| *COMT* rs4680 | |  |  |  |  |  |  |  |  |  |  |  |  |  |  |  |
|  | AA | 64.0 | 81 | 32 |  | 61.1 | 80 | 32 |  | 52.0 | 54 | 8 |  | 52.9 | 81 | 46 |
|  | AG | 62.3 | 78 | 28 |  | 61.3 | 79 | 27 |  | 52.0 | 75 | 31 |  | 52.8 | 83 | 27 |
|  | GG | 54.8 | 84 | 25 |  | 61.5 | 80 | 43 |  | 42.9 | 74 | 24 |  | 50.0 | 87 | 48 |
|  | Kruskal-Wallis ANOVA | *H* = 2.98; *p* = 0.225 | | |  | *H* = 1.17; *p* = 0.557 | | |  | *H* = 1.88; *p* = 0.391 | | |  | *H* = 0.10; *p* = 0.952 | | |
| PANSS_0-6_ negative symptom scores reduction after 8 weeks of treatment | | | | | | | | | | | | | | | | |
| *COMT* rs4680 | |  |  |  |  |  |  |  |  |  |  |  |  |  |  |  |
|  | AA | 33.3 | 80 | 33 |  | 35.7 | 89 | 42 |  | 28.9 | 37 | 6 |  | 30.4 | 86 | 56 |
|  | AG | 43.7 | 86 | 36 |  | 44.8 | 189 | 37 |  | 27.7 | 64 | 20 |  | 35.6 | 86 | 48 |
|  | GG | 36.4 | 96 | 33 |  | 29.2 | 74 | 29 |  | 25.0 | 44 | 16 |  | 35.7 | 77 | 40 |
|  | Kruskal-Wallis ANOVA | *H* = 3.68; *p* = 0.160 | | |  | *H* = 1.54; *p* = 0.463 | | |  | *H* = 0.32; *p* = 0.853 | | |  | *H* = 0.29; *p* = 0.864 | | |
| PANSS_0-6_ general psychopathology scores reduction after 8 weeks of treatment | | | | | | | | | | | | | | | | |
| *COMT* rs4680 | |  |  |  |  |  |  |  |  |  |  |  |  |  |  |  |
|  | AA | 51.2 | 94 | 37 |  | 46.7 | 84 | 26 |  | 50.7 | 74 | 21 |  | 41.7 | 58 | 31 |
|  | AG | 57.0 | 93 | 25 |  | 59.6 | 78 | 33 |  | 45.4 | 74 | 21 |  | 51.6 | 83 | 30 |
|  | GG | 47.8 | 72 | 19 |  | 48.6 | 78 | 51 |  | 43.2 | 46 | 22 |  | 38.5 | 84 | 42 |
|  | Kruskal-Wallis ANOVA | *H* = 5.01; *p* = 0.082 | | |  | *H* = 1.36; *p* = 0.508 | | |  | *H* = 1.12; *p* = 0.570 | | |  | *H* = 0.87; *p* = 0.648 | | |

Values are given as median, range and interquartile range (IR); *n* = number of subjects; PANSS = Positive and Negative Syndrome Scale.

Supplementary Table S4. Percentage reduction from the initial PANSS_0-6_ total and subscale scores after 8 weeks of treatment with olanzapine, risperidone, clozapine or other antipsychotics in male schizophrenic patients subdivided according to the *COMT* rs4818 genotypes.

|  | | Olanzapine  *n* = 144 | | |  | Risperidone  *n* = 69 | | |  | Clozapine  *n* = 71 | | |  | Other antipsychotics  *n* = 68 | | |
| --- | --- | --- | --- | --- | --- | --- | --- | --- | --- | --- | --- | --- | --- | --- | --- | --- |
|  |  | Median | Range | IR |  | Median | Range | IR |  | Median | Range | IR |  | Median | Range | IR |
| Total PANSS_0-6_ scores reduction after 8 weeks of treatment | | | | | | | | | | | | | | | | |
| *COMT* genotype | |  |  |  |  |  |  |  |  |  |  |  |  |  |  |  |
|  | CC | 49.4 | 83 | 27 |  | 48.6 | 79 | 30 |  | 47.1 | 46 | 20 |  | 48.7 | 66 | 33 |
|  | CG | 59.6 | 81 | 23 |  | 57.9 | 70 | 18 |  | 46.0 | 61 | 17 |  | 48.8 | 78 | 37 |
|  | GG | 49.5 | 60 | 21 |  | 38.4 | 72 | 41 |  | 43.0 | 46 | 27 |  | 39.8 | 71 | 47 |
|  | Kruskal-Wallis ANOVA | *H* = 8.16; *p* = 0.017 | | |  | *H* = 1.62; *p* = 0.445 | | |  | *H* = 0.47; *p* = 0.790 | | |  | *H* = 0.05; *p* = 0.978 | | |
| PANSS_0-6_ positive symptom scores reduction after 8 weeks of treatment | | | | | | | | | | | | | | | | |
| *COMT* genotype | |  |  |  |  |  |  |  |  |  |  |  |  |  |  |  |
|  | CC | 60.0 | 82 | 28 |  | 61.1 | 81 | 31 |  | 51.7 | 54 | 20 |  | 52.3 | 82 | 34 |
|  | CG | 66.7 | 88 | 27 |  | 67.5 | 77 | 26 |  | 52.0 | 65 | 25 |  | 52.3 | 86 | 32 |
|  | GG | 51.6 | 76 | 22 |  | 48.4 | 72 | 40 |  | 46.6 | 74 | 27 |  | 51.5 | 85 | 58 |
|  | Kruskal-Wallis ANOVA | *H* = 6.92; *p* = 0.031 | | |  | *H* = 2.73; *p* = 0.256 | | |  | *H* = 2.09; *p* = 0.352 | | |  | *H* = 0.14; *p* = 0.931 | | |
| PANSS_0-6_ negative symptom scores reduction after 8 weeks of treatment | | | | | | | | | | | | | | | | |
| *COMT* genotype | |  |  |  |  |  |  |  |  |  |  |  |  |  |  |  |
|  | CC | 33.3 | 80 | 33 |  | 29.2 | 89 | 37 |  | 26.3 | 37 | 9 |  | 35.3 | 86 | 36 |
|  | CG | 42.9 | 104 | 37 |  | 44.4 | 189 | 41 |  | 28.4 | 64 | 19 |  | 40.4 | 85 | 48 |
|  | GG | 36.1 | 79 | 28 |  | 30.7 | 74 | 33 |  | 30.6 | 44 | 20 |  | 23.7 | 69 | 57 |
|  | Kruskal-Wallis ANOVA | *H* = 2.12; *p* = 0.347 | | |  | *H* = 0.67; *p* = 0.716 | | |  | *H* = 1.38; *p* = 0.502 | | |  | *H* = 0.42; *p* = 0.810 | | |
| PANSS_0-6_ general psychopathology scores reduction after 8 weeks of treatment | | | | | | | | | | | | | | | | |
| *COMT* genotype | |  |  |  |  |  |  |  |  |  |  |  |  |  |  |  |
|  | CC | 46.8 | 90 | 31 |  | 46.7 | 84 | 30 |  | 46.7 | 68 | 19 |  | 46.3 | 66 | 31 |
|  | CG | 59.3 | 93 | 21 |  | 60.1 | 78 | 25 |  | 45.4 | 66 | 21 |  | 49.5 | 83 | 36 |
|  | GG | 50.5 | 63 | 14 |  | 41.7 | 78 | 50 |  | 44.0 | 46 | 26 |  | 39.4 | 76 | 49 |
|  | Kruskal-Wallis ANOVA | *H* = 8.74; *p* = 0.013 | | |  | *H* = 1.83; *p* = 0.400 | | |  | *H* = 0.21; *p* = 0.902 | | |  | *H* = 0.30; *p* = 0.860 | | |

Values are given as median, range and interquartile range (IR); *n* = number of subjects; PANSS = Positive and Negative Syndrome Scale.

Supplementary Table S5. Haplotype frequencies of *COMT* rs4680 and rs4818 polymorphisms in male schizophrenia patients treated with olanzapine, risperidone, clozapine or other antipsychotics, subdivided into responders (R) and non-responders (NR) according to the 50% reduction in PANSS_0-6_ total and subscale scores.

| *COMT* rs4680-rs4818 |  | Olanzapine  *n* = 144 | |  | Risperidone  *n* = 69 | |  | Clozapine  *n* = 71 | |  | Other antipsychotics  *n* = 68 | |
| --- | --- | --- | --- | --- | --- | --- | --- | --- | --- | --- | --- | --- |
| C-A haplotype carriers |  | C-A carriers | Non-carriers |  | C-A carriers | Non-carriers |  | C-A carriers | Non-carriers |  | C-A carriers | Non-carriers |
| Total PANSS_0-6_ scores reduction at week 8 | NR | 40  (67.8) | 19  (32.2) |  | 21  (70.0) | 9  (30.0) |  | 33  (73.3) | 12  (26.7) |  | 24  (66.7) | 12  (33.3) |
|  | R | 68  (80.0) | 17  (20.0) |  | 31  (79.5) | 8  (20.5) |  | 21  (80.8) | 5  (19.2) |  | 25  (78.1) | 7  (21.9) |
|  |  | *χ^2^* = 2.77; *p* = 0.096 | |  | *χ^2^* = 0.82; *p* = 0.365 | |  | *χ^2^* = 0.50; *p* = 0.479 | |  | *χ^2^* = 1.11; *p* = 0.293 | |
| PANSS_0-6_ positive scores reduction at week 8 | NR | 25  (64.1) | 14  (35.9) |  | 15  (68.2) | 7  (31.8) |  | 19  (63.3) | 11  (36.7) |  | 18  (66.7) | 9  (33.3) |
|  | R | 83  (79.0) | 22  (21.0) |  | 37  (78.7) | 10  (21.3) |  | 35  (85.4) | 6  (14.6) |  | 31  (75.6) | 10  (24.4) |
|  |  | *χ^2^* = 3.39; *p* = 0.066 | |  | *χ^2^* = 0.90; *p* = 0.344 | |  | *χ^2^* = 4.62; *p* = 0.032 | |  | *χ^2^* = 0.65; *p* = 0.421 | |
| PANSS_0-6_ negative scores reduction at week 8 | NR | 67  (71.3) | 27  (28.7) |  | 32  (71.1) | 13  (28.9) |  | 49  (74.2) | 17  (25.8) |  | 33  (68.8) | 15  (31.3) |
|  | R | 41  (82.0) | 9  (18.0) |  | 20  (83.3) | 4  (16.7) |  | 5  (100.0) | 0  (0.0) |  | 16  (80.0) | 4  (20.0) |
|  |  | *χ^2^* = 2.00; *p* = 0.157 | |  | *χ^2^* = 1.26; *p* = 0.262 | |  | *χ^2^* = 1.69; *p* = 0.193 | |  | *χ^2^* = 0.89; *p* = 0.346 | |
| PANSS_0-6_ general psychopathology scores reduction at week 8 | NR | 39  (69.6) | 17  (30.4) |  | 20  (66.7) | 10  (33.3) |  | 31  (73.8) | 11  (26.2) |  | 23  (65.7) | 12  (34.3) |
|  | R | 69  (78.4) | 19  (21.6) |  | 32  (82.1) | 7  (17.9) |  | 23  (79.3) | 6  (20.7) |  | 26  (78.8) | 7  (21.2) |
|  |  | *χ^2^* = 1.40; *p* = 0.236 | |  | *χ^2^* = 2.16; *p* = 0.142 | |  | *χ^2^* = 0.29; *p* = 0.593 | |  | *χ^2^* = 1.44; *p* = 0.230 | |

Frequencies (%) are shown in parenthesis; *n* = number of subjects; NR = non-responders; PANSS = Positive and Negative Syndrome Scale; R = responders.

Supplementary Table S6. Percentage reduction from the initial PANSS_0-6_ total and subscale scores after 8 weeks of treatment with olanzapine, risperidone, clozapine or other antipsychotics in male schizophrenic patients subdivided according to the *COMT* rs4680-rs4818 haplotypes into C-A haplotype carriers and carriers of the other haplotypes (non-carriers).

|  | | Olanzapine  *n* = 144 | | |  | Risperidone  *n* = 69 | | |  | Clozapine  *n* = 71 | | |  | Other antipsychotics  *n* = 68 | | |
| --- | --- | --- | --- | --- | --- | --- | --- | --- | --- | --- | --- | --- | --- | --- | --- | --- |
|  |  | Median | Range | IR |  | Median | Range | IR |  | Median | Range | IR |  | Median | Range | IR |
| Total PANSS_0-6_ scores reduction after 8 weeks of treatment | | | | | | | | | | | | | | | | |
| *COMT* rs4680-rs4818 | |  |  |  |  |  |  |  |  |  |  |  |  |  |  |  |
|  | C-A carriers | 56.9 | 86 | 26 |  | 56.3 | 81 | 30 |  | 47.4 | 66 | 14 |  | 50.0 | 84 | 33 |
|  | Non-carriers | 49.5 | 71 | 26 |  | 49.4 | 72 | 40 |  | 41.7 | 46 | 22 |  | 43.4 | 79 | 41 |
|  | Mann-Whitney U test | *U* = 1456.5; *p* = 0.025 | | |  | *U* = 372.0; *p* = 0.330 | | |  | *U* = 385.0; *p* = 0.319 | | |  | *U* = 426.0; *p* = 0.589 | | |
| PANSS_0-6_ positive symptom scores reduction after 8 weeks of treatment | | | | | | | | | | | | | | | | |
| *COMT* rs4680-rs4818 | |  |  |  |  |  |  |  |  |  |  |  |  |  |  |  |
|  | C-A carriers | 63.1 | 81 | 28 |  | 61.2 | 83 | 30 |  | 52.0 | 76 | 19 |  | 52.9 | 86 | 33 |
|  | Non-carriers | 53.4 | 84 | 26 |  | 61.5 | 80 | 43 |  | 42.9 | 74 | 24 |  | 50.0 | 87 | 51 |
|  | Mann-Whitney U test | *U* = 1522.0; *p* = 0.052 | | |  | *U* = 367.5; *p* = 0.299 | | |  | *U* = 357.5; *p* = 0.171 | | |  | *U* = 423.0; *p* = 0.561 | | |
| PANSS_0-6_ negative symptom scores reduction after 8 weeks of treatment | | | | | | | | | | | | | | | | |
| *COMT* rs4680-rs4818 | |  |  |  |  |  |  |  |  |  |  |  |  |  |  |  |
|  | C-A carriers | 43.2 | 86 | 37 |  | 39.7 | 198 | 42 |  | 28.6 | 64 | 12 |  | 35.3 | 86 | 48 |
|  | Non-carriers | 36.1 | 96 | 36 |  | 29.2 | 74 | 29 |  | 25.0 | 44 | 16 |  | 35.7 | 77 | 29 |
|  | Mann-Whitney U test | *U* = 1543.0; *p* = 0.064 | | |  | *U* = 402.0; *p* = 0.577 | | |  | *U* = 417.5; *p* = 0.576 | | |  | *U* = 488.5; *p* = 0.753 | | |
| PANSS_0-6_ general psychopathology scores reduction after 8 weeks of treatment | | | | | | | | | | | | | | | | |
| *COMT* rs4680-rs4818 | |  |  |  |  |  |  |  |  |  |  |  |  |  |  |  |
|  | C-A carriers | 56.4 | 94 | 26 |  | 57.7 | 87 | 30 |  | 46.7 | 74 | 19 |  | 50.0 | 83 | 29 |
|  | Non-carriers | 50.5 | 72 | 24 |  | 48.6 | 78 | 51 |  | 43.2 | 46 | 22 |  | 38.5 | 84 | 35 |
|  | Mann-Whitney U test | *U* = 1563.5; *p* = 0.079 | | |  | *U* = 374.5; *p* = 0.347 | | |  | *U* = 457.0; *p* = 0.978 | | |  | *U* = 407.5; *p* = 0.428 | | |

Values are given as median. range and interquartile range (IR); *n* = number of subjects; PANSS = Positive and Negative Syndrome Scale.

Supplementary Table S7. The *COMT* rs4680 genotype count and frequencies in female schizophrenia patients treated with olanzapine, risperidone, clozapine or other antipsychotics, subdivided into responders (R) and non-responders (NR) according to the 50% reduction in the baseline PANSS_0-6_ total and subscale scores.

|  |  | Olanzapine  *n* = 46 | | |  | Risperidone  *n* = 30 | | |  | Clozapine  *n* = 31 | | |  | Other antipsychotics  *n* = 62 | | |
| --- | --- | --- | --- | --- | --- | --- | --- | --- | --- | --- | --- | --- | --- | --- | --- | --- |
| *COMT* rs4680 |  | AA | AG | GG |  | AA | AG | GG |  | AA | AG | GG |  | AA | AG | GG |
| Total PANSS_0-6_ score reduction at week 8 | NR | 3 (17.6) | 8  (47.1) | 6 (35.3) |  | 1  (12.5) | 6  (75.0) | 1 (12.5) |  | 4 (44.4) | 2 (22.2) | 3 (33.3) |  | 3 (25.0) | 5 (41.7) | 4 (33.3) |
|  | R | 8 (27.6) | 17 (58.6) | 4 (13.8) |  | 2 (9.1) | 11 (50.0) | 9 (40.9) |  | 6 (27.3) | 8 (36.4) | 8 (36.4) |  | 10 (20.0) | 26 (52.0) | 14 (28.0) |
|  |  | *χ ^2^* = 2.99; *p* =0.225 | | |  | *χ^2^* = 2.14; *p* = 0.344 | | |  | *χ^2^* = 1.00; *p* = 0.608 | | |  | *χ^2^* = 0.42; *p* = 0.812 | | |
| PANSS_0-6_ positive scores reduction at week 8 | NR | 2 (15.4) | 6 (46.2) | 5 (38.5) |  | 1  (16.7) | 5 (83.3) | 0 (0.0) |  | 4 (66.7) | 1 (16.7) | 1 (16.7) |  | 3  (25.0) | 5 (41.7) | 4 (33.3) |
|  | R | 9  (27.3) | 19 (57.6) | 5 (15.2) |  | 2 (8.3) | 12 (50.0) | 10 (41.7) |  | 6 (24.0) | 9 (36.0) | 10 (40.0) |  | 10 (20.0) | 26 (52.0) | 14 (28.0) |
|  |  | *χ^2^* = 3.11; *p* = 0.212 | | |  | *χ^2^* = 3.78; *p* = 0.151 | | |  | *χ^2^* = 4.03; *p* = 0.133 | | |  | *χ^2^* = 0.42; *p* = 0.812 | | |
| PANSS_0-6_ negative scores reduction at week 8 | NR | 7 (24.1) | 13  (44.8) | 9 (31.0) |  | 1 (10.0) | 7 (70.0) | 2 (20.0) |  | 6 (28.6) | 8 (38.1) | 7 (33.3) |  | 4 (19.0) | 10 (47.6) | 7 (33.3) |
|  | R | 4 (23.5) | 12 (70.6) | 1 (5.9) |  | 2 (10.0) | 10 (50.0) | 8 (40.0) |  | 4 (40.0) | 2 (20.0) | 4 (40.0) |  | 9 (22.0) | 21 (51.2) | 11 (26.8) |
|  |  | *χ^2^* = 4.43; *p* = 0.109 | | |  | *χ^2^* = 1.27; *p* = 0.530 | | |  | *χ^2^* = 1.05; *p* = 0.593 | | |  | *χ^2^* = 0.29; *p* = 0.863 | | |
| PANSS_0-6_ general psychopathology scores reduction at week 8 | NR | 5 (18.5) | 13 (48.1) | 9 (33.3) |  | 1 (8.3) | 9  (75.0) | 2 (16.7) |  | 4 (26.7) | 5 (33.3) | 6 (40.0) |  | 7 (29.2) | 10 (41.7) | 7 (29.2) |
|  | R | 6  (31.6) | 12 (63.2) | 1 (5.3) |  | 2 (11.1) | 8 (44.4) | 8 (44.4) |  | 6  (37.5) | 5 (31.3) | 5 (31.3) |  | 6 (15.8) | 21 (55.3) | 11 (28.9) |
|  |  | *χ^2^* = 5.30; *p* = 0.071 | | |  | *χ^2^* = 2.91; *p* = 0.234 | | |  | *χ^2^* = 0.46; *p* = 0.795 | | |  | *χ^2^* = 1.80; *p* = 0.407 | | |

Frequencies (%) are shown in parenthesis; *n* = number of subjects; NR = non-responders; PANSS = Positive and Negative Syndrome Scale; R = responders.

Supplementary Table S8. The *COMT* rs4818 genotype count and frequencies in female schizophrenia patients treated with olanzapine, risperidone, clozapine or other antipsychotics, subdivided into responders (R) and non-responders (NR) according to the 50% reduction in PANSS_0-6_ total and subscale scores.

|  |  | Olanzapine  *n* = 46 | | |  | Risperidone  *n* = 30 | | |  | Clozapine  *n* = 31 | | |  | Other antipsychotics  *n* = 62 | | |
| --- | --- | --- | --- | --- | --- | --- | --- | --- | --- | --- | --- | --- | --- | --- | --- | --- |
| *COMT* rs4818 |  | CC | GC | GG |  | CC | GC | GG |  | CC | GC | GG |  | CC | GC | GG |
| Total PANSS_0-6_ scores reduction at week 8 | NR | 5 (29.4) | 8 (47.1) | 4 (23.5) |  | 2 (25.0) | 6  (75.0) | 0 (0.0) |  | 1 (11.1) | 7 (77.8) | 1 (11.1) |  | 6 (50.0) | 3 (25.0) | 3 (25.0) |
|  | R | 13 (44.8) | 12 (41.4) | 4 (13.8) |  | 7 (31.8) | 10 (45.5) | 5 (22.7) |  | 7 (31.8) | 11 (50.0) | 4 (18.2) |  | 20 (40.0) | 22 (44.0) | 8 (16.0) |
|  |  | *χ ^2^* = 1.32; *p* =0.518 | | |  | *χ^2^* = 2.87; *p* = 0.238 | | |  | *χ^2^* = 2.11; *p* = 0.349 | | |  | *χ^2^* = 1.54; *p* = 0.463 | | |
| PANSS_0-6_ positive scores reduction at week 8 | NR | 3  (23.1) | 8 (61.5) | 2  (15.4) |  | 1 (16.7) | 5 (83.3) | 0 (0.0) |  | 1 (16.7) | 4 (66.7) | 1 (16.7) |  | 6  (50.0) | 5  (41.7) | 1  (8.3) |
|  | R | 15 (45.5) | 12 (36.4) | 6 (18.2) |  | 8  (33.3) | 11 (45.8) | 5 (20.8) |  | 7 (28.0) | 14 (56.0) | 4 (16.0) |  | 20 (40.0) | 20 (40.0) | 10 (20.0) |
|  |  | *χ^2^* = 2.60; *p* = 0.273 | | |  | *χ^2^* = 2.96; *p* = 0.228 | | |  | *χ^2^* = 0.34; *p* = 0.845 | | |  | *χ^2^* = 0.98; *p* = 0.613 | | |
| PANSS_0-6_ negative scores reduction at week 8 | NR | 11 (37.9) | 12 (41.4) | 6 (20.7) |  | 3 (30.0) | 7  (70.0) | 0 (0.0) |  | 4 (19.0) | 15 (71.4) | 2 (9.5) |  | 9 (42.9) | 9 (42.9) | 3 (14.3) |
|  | R | 7  (41.2) | 8 (47.1) | 2 (11.8) |  | 6 (30.0) | 9 (45.0) | 5 (25.0) |  | 4  (40.0) | 3 (3.0) | 3 (3.0) |  | 17 (41.5) | 16 (39.0) | 8 (19.5) |
|  |  | *χ^2^* = 0.60; *p* = 0.741 | | |  | *χ^2^* = 3.28; *p* = 0.194 | | |  | *χ^2^* = 4.92; *p* = 0.086 | | |  | *χ^2^* = 0.27; *p* = 0.873 | | |
| PANSS_0-6_ general psychopathology scores reduction at week 8 | NR | 7  (25.9) | 13 (48.1) | 7 (25.9) |  | 3 (25.0) | 8 (66.7) | 1 (8.3) |  | 1 (6.7) | 12 (80.0) | 2 (13.3) |  | 12  (50.0) | 7 (29.2) | 5 (20.8) |
|  | R | 11 (57.9) | 7 (36.8) | 1 (5.3) |  | 6 (33.3) | 8 (44.4) | 4 (22.2) |  | 7 (43.8) | 6 (37.5) | 3 (18.8) |  | 14 (36.8) | 18 (47.4) | 6 (15.8) |
|  |  | *χ^2^* = 5.98; *p* = 0.050 | | |  | *χ^2^* = 1.67; *p* = 0.435 | | |  | *χ^2^* = 6.68; *p* = 0.036 | | |  | *χ^2^* = 2.03; *p* = 0.363 | | |

Frequencies (%) are shown in parenthesis; *n* = number of subjects; NR = non-responders; PANSS = Positive and Negative Syndrome Scale; R = responders.

Supplementary Table S9. Percentage of reduction from the initial PANSS_0-6_ total and subscale scores after 8 weeks of treatment with olanzapine, risperidone, clozapine or other antipsychotics in female schizophrenia patients subdivided according to the *COMT* rs4680 genotypes.

|  | | Olanzapine  *n* = 46 | | |  | Risperidone  *n* = 30 | | |  | Clozapine  *n* = 31 | | |  | Other antipsychotics  *n* = 62 | | |
| --- | --- | --- | --- | --- | --- | --- | --- | --- | --- | --- | --- | --- | --- | --- | --- | --- |
|  |  | Median | Range | IR |  | Median | Range | IR |  | Median | Range | IR |  | Median | Range | IR |
| Total PANSS_0-6_ scores reduction after 8 weeks of treatment | | | | | | | | | | | | | | | | |
| *COMT* rs4680 | |  |  |  |  |  |  |  |  |  |  |  |  |  |  |  |
|  | AA | 66.7 | 59 | 34 |  | 68.8 | 63 | 0 |  | 63.6 | 49 | 43 |  | 58.5 | 83 | 27 |
|  | AG | 60.0 | 65 | 24 |  | 59.6 | 56 | 27 |  | 65.9 | 48 | 20 |  | 66.7 | 57 | 16 |
|  | GG | 46.4 | 58 | 16 |  | 67.9 | 39 | 12 |  | 62.9 | 35 | 23 |  | 61.2 | 48 | 22 |
|  | Kruskal-Wallis ANOVA | *H* = 4.08; *p* = 0.130 | | |  | *H* = 2.09; *p* = 0.352 | | |  | *H* = 1.07; *p* = 0.587 | | |  | *H* = 2.54; *p* = 0.281 | | |
| PANSS_0-6_ positive symptom scores reduction after 8 weeks of treatment | | | | | | | | | | | | | | | | |
| *COMT* rs4680 | |  |  |  |  |  |  |  |  |  |  |  |  |  |  |  |
|  | AA | 71.4 | 67 | 33 |  | 66.7 | 61 | 0 |  | 74.5 | 62 | 46 |  | 68.8 | 78 | 40 |
|  | AG | 75.0 | 79 | 36 |  | 61.5 | 80 | 37 |  | 82.3 | 55 | 19 |  | 73.7 | 70 | 23 |
|  | GG | 49.2 | 62 | 31 |  | 71.9 | 32 | 17 |  | 66.7 | 49 | 28 |  | 80.1 | 62 | 38 |
|  | Kruskal-Wallis ANOVA | *H* = 3.62; *p* = 0.164 | | |  | *H* = 2.36; *p* = 0.307 | | |  | *H* = 3.28; *p* = 0.194 | | |  | *H* = 0.55; *p* = 0.760 | | |
| PANSS_0-6_ negative symptom scores reduction after 8 weeks of treatment | | | | | | | | | | | | | | | | |
| *COMT* rs4680 | |  |  |  |  |  |  |  |  |  |  |  |  |  |  |  |
|  | AA | 43.8 | 59 | 46 |  | 80.0 | 80 | 0 |  | 35.4 | 68 | 41 |  | 56.3 | 88 | 48 |
|  | AG | 38.5 | 86 | 43 |  | 63.2 | 80 | 40 |  | 35.4 | 67 | 36 |  | 60.0 | 82 | 35 |
|  | GG | 35.1 | 57 | 21 |  | 69.2 | 53 | 26 |  | 40.0 | 58 | 35 |  | 53.1 | 66 | 34 |
|  | Kruskal-Wallis ANOVA | *H* = 1.38; *p* = 0.503 | | |  | *H* = 2.22; *p* = 0.330 | | |  | *H* = 0.55; *p* = 0.760 | | |  | *H* = 0.59; *p* = 0.744 | | |
| PANSS_0-6_ general psychopathology scores reduction after 8 weeks of treatment | | | | | | | | | | | | | | | | |
| *COMT* rs4680 | |  |  |  |  |  |  |  |  |  |  |  |  |  |  |  |
|  | AA | 55.2 | 77 | 24 |  | 56.5 | 58 | 0 |  | 55.8 | 45 | 32 |  | 48.1 | 83 | 34 |
|  | AG | 46.7 | 65 | 25 |  | 47.1 | 77 | 21 |  | 47.2 | 62 | 37 |  | 57.7 | 79 | 29 |
|  | GG | 44.7 | 50 | 12 |  | 58.6 | 47 | 24 |  | 44.6 | 41 | 31 |  | 51.1 | 89 | 20 |
|  | Kruskal-Wallis ANOVA | *H* = 3.01; *p* = 0.222 | | |  | *H* = 3.37; *p* = 0.185 | | |  | *H* = 0.33; *p* = 0.846 | | |  | *H* = 3.00; *p* = 0.224 | | |

Values are given as median, range and interquartile range (IR); *n* = number of subjects; PANSS = Positive and Negative Syndrome Scale.

Supplementary Table S10. Percentage reduction from the initial PANSS_0-6_ total and subscale scores after 8 weeks of treatment with olanzapine, risperidone, clozapine or other antipsychotics in female schizophrenic patients subdivided according to the *COMT* rs4818 genotypes.

|  | | Olanzapine  *n* = 46 | | |  | Risperidone  *n* = 30 | | |  | Clozapine  *n* = 31 | | |  | Other antipsychotics  *n* = 62 | | |
| --- | --- | --- | --- | --- | --- | --- | --- | --- | --- | --- | --- | --- | --- | --- | --- | --- |
|  |  | Median | Range | IR |  | Median | Range | IR |  | Median | Range | IR |  | Median | Range | IR |
| Total PANSS_0-6_ scores reduction after 8 weeks of treatment | | | | | | | | | | | | | | | | |
| *COMT* genotype | |  |  |  |  |  |  |  |  |  |  |  |  |  |  |  |
|  | CC | 66.9 | 59 | 29 |  | 68.5 | 55 | 23 |  | 69.6 | 45 | 9 |  | 60.8 | 83 | 17 |
|  | CG | 56.2 | 58 | 30 |  | 61.5 | 68 | 27 |  | 60.2 | 55 | 29 |  | 68.0 | 57 | 19 |
|  | GG | 50.0 | 59 | 19 |  | 74.3 | 18 | 15 |  | 64.8 | 30 | 18 |  | 67.2 | 39 | 26 |
|  | Kruskal-Wallis ANOVA | *H* = 4.24; *p* = 0.120 | | |  | *H* = 5.43; *p* = 0.066 | | |  | *H* = 2.73; *p* = 0.255 | | |  | *H* = 2.61; *p* = 0.271 | | |
| PANSS_0-6_ positive symptom scores reduction after 8 weeks of treatment | | | | | | | | | | | | | | | | |
| *COMT* genotype | |  |  |  |  |  |  |  |  |  |  |  |  |  |  |  |
|  | CC | 74.4 | 71 | 28 |  | 66.7 | 80 | 26 |  | 82.0 | 62 | 10 |  | 70.5 | 78 | 34 |
|  | CG | 67.5 | 79 | 48 |  | 61.5 | 80 | 27 |  | 74.4 | 66 | 35 |  | 73.9 | 70 | 28 |
|  | GG | 56.3 | 62 | 30 |  | 78.6 | 21 | 13 |  | 62.5 | 49 | 38 |  | 83.3 | 51 | 26 |
|  | Kruskal-Wallis ANOVA | *H* = 1.48; *p* = 0.477 | | |  | *H* = 5.35; *p* = 0.069 | | |  | *H* = 0.51; *p* = 0.777 | | |  | *H* = 2.30; *p* = 0.316 | | |
| PANSS_0-6_ negative symptom scores reduction after 8 weeks of treatment | | | | | | | | | | | | | | | | |
| *COMT* genotype | |  |  |  |  |  |  |  |  |  |  |  |  |  |  |  |
|  | CC | 41.4 | 86 | 47 |  | 64.7 | 66 | 39 |  | 47.8 | 57 | 41 |  | 56.7 | 88 | 36 |
|  | CG | 38.8 | 70 | 33 |  | 65.5 | 80 | 40 |  | 31.7 | 67 | 29 |  | 64.3 | 82 | 37 |
|  | GG | 34.5 | 57 | 38 |  | 78.9 | 27 | 19 |  | 50.0 | 52 | 38 |  | 53.3 | 66 | 29 |
|  | Kruskal-Wallis ANOVA | *H* = 1.40; *p* = 0.497 | | |  | *H* = 3.16; *p* = 0.207 | | |  | *H* = 4.18; *p* = 0.124 | | |  | *H* = 1.31; *p* = 0.521 | | |
| PANSS_0-6_ general psychopathology scores reduction after 8 weeks of treatment | | | | | | | | | | | | | | | | |
| *COMT* genotype | |  |  |  |  |  |  |  |  |  |  |  |  |  |  |  |
|  | CC | 56.0 | 77 | 25 |  | 56.5 | 48 | 30 |  | 64.0 | 34 | 12 |  | 51.9 | 83 | 33 |
|  | CG | 41.8 | 56 | 21 |  | 48.6 | 77 | 21 |  | 35.7 | 62 | 31 |  | 57.6 | 73 | 19 |
|  | GG | 46.0 | 58 | 4 |  | 71.0 | 31 | 29 |  | 58.1 | 28 | 22 |  | 50.0 | 89 | 36 |
|  | Kruskal-Wallis ANOVA | *H* = 5.26; *p* = 0.072 | | |  | *H* = 3.52; *p* = 0.172 | | |  | *H* = 5.05; *p* = 0.080 | | |  | *H* = 1.85; *p* = 0.397 | | |

Values are given as median, range and interquartile range (IR); *n* = number of subjects; PANSS = Positive and Negative Syndrome Scale.

Supplementary Table S11. Haplotype frequencies of *COMT* rs4680 and rs4818 polymorphisms in female schizophrenia patients treated with olanzapine, risperidone, clozapine or other antipsychotics, subdivided into responders (R) and non-responders (NR) according to the 50% reduction in PANSS_0-6_ total and subscale scores.

| *COMT* rs4680-rs4818 |  | Olanzapine  *n* = 46 | |  | Risperidone  *n* = 30 | |  | Clozapine  *n* = 31 | |  | Other antipsychotics  *n* = 62 | |
| --- | --- | --- | --- | --- | --- | --- | --- | --- | --- | --- | --- | --- |
| C-A haplotype carriers |  | C-A carriers | Non-carriers |  | C-A carriers | Non-carriers |  | C-A carriers | Non-carriers |  | C-A carriers | Non-carriers |
| Total PANSS_0-6_ scores reduction at week 8 | NR | 11  (64.7) | 6  (35.3) |  | 7  (87.5) | 1  (12.5) |  | 6  (66.7) | 3  (33.3) |  | 8  (66.7) | 4  (33.3) |
|  | R | 24  (82.8) | 5  (17.2) |  | 13  (59.1) | 9  (40.9) |  | 14  (63.6) | 8  (36.4) |  | 35  (70.0) | 15  (30.0) |
|  |  | *χ^2^* = 1.92; *p* = 0.166 | |  | *χ^2^* = 2.13; *p* = 0.144 | |  | *χ^2^* = 0.03; *p* = 0.873 | |  | *χ^2^* = 0.05; *p* = 0.822 | |
| PANSS_0-6_ positive scores reduction at week 8 | NR | 8  (61.5) | 5  (38.5) |  | 6  (100.0) | 0  (0.0) |  | 5  (83.3) | 1  (16.7) |  | 8  (66.7) | 4  (33.3) |
|  | R | 27  (81.8) | 6  (18.2) |  | 14  (58.3) | 10  (41.7) |  | 15  (60.0) | 10  (40.0) |  | 35  (70.0) | 15  (30.0) |
|  |  | *χ^2^* = 2.11; *p* = 0.147 | |  | *χ^2^* = 3.75; *p* = 0.053 | |  | *χ^2^* = 1.15; *p* = 0.283 | |  | *χ^2^* = 0.05; *p* = 0.822 | |
| PANSS_0-6_ negative scores reduction at week 8 | NR | 20  (69.0) | 9  (31.0) |  | 38  (80.0) | 2  (20.0) |  | 14  (66.7) | 7  (33.3) |  | 14  (66.7) | 7  (33.3) |
|  | R | 15  (88.2) | 2  (11.8) |  | 12  (60.0) | 8  (40.0) |  | 6  (60.0) | 4  (40.0) |  | 29  (70.7) | 12  (29.3) |
|  |  | *χ^2^* = 2.19; *p* = 0.139 | |  | *χ^2^* = 1.20; *p* = 0.273 | |  | *χ^2^* = 0.13; *p* = 0.717 | |  | *χ^2^* = 0.11; *p* = 0.742 | |
| PANSS_0-6_ general psychopathology scores reduction at week 8 | NR | 18  (66.7) | 9  (33.3) |  | 10  (83.3) | 2  (16.7) |  | 9  (60.0) | 6  (40.0) |  | 17  (70.8) | 7  (29.2) |
|  | R | 17  (89.5) | 2  (10.5) |  | 10  (55.6) | 8  (44.4) |  | 11  (68.8) | 5  (31.3) |  | 26  (68.4) | 12  (31.6) |
|  |  | *χ^2^* = 3.19; *p* = 0.074 | |  | *χ^2^* = 2.50; *p* = 0.114 | |  | *χ^2^* = 0.26; *p* = 0.611 | |  | *χ^2^* = 0.04; *p* = 0.841 | |

Frequencies (%) are shown in parenthesis; *n* = number of subjects; NR = non-responders; PANSS = Positive and Negative Syndrome Scale; R = responders.

Supplementary Table S12. Percentage reduction from the initial PANSS_0-6_ total and subscale scores after 8 weeks of treatment with olanzapine, risperidone, clozapine or other antipsychotics in female schizophrenic patients subdivided according to the *COMT* rs4680-rs4818 haplotypes into C-A haplotype carriers and carriers of the other haplotypes (non-carriers).

|  | | Olanzapine  *n* = 46 | | |  | Risperidone  *n* = 30 | | |  | Clozapine  *n* = 31 | | |  | Other antipsychotics  *n* = 62 | | |
| --- | --- | --- | --- | --- | --- | --- | --- | --- | --- | --- | --- | --- | --- | --- | --- | --- |
|  |  | Median | Range | IR |  | Median | Range | IR |  | Median | Range | IR |  | Median | Range | IR |
| Total PANSS_0-6_ scores reduction after 8 weeks of treatment | | | | | | | | | | | | | | | | |
| *COMT* rs4680-rs4818 | |  |  |  |  |  |  |  |  |  |  |  |  |  |  |  |
|  | C-A carriers | 60.0 | 72 | 27 |  | 61.5 | 68 | 28 |  | 64.9 | 55 | 33 |  | 65.3 | 83 | 18 |
|  | Non-carriers | 47.8 | 59 | 17 |  | 67.9 | 39 | 12 |  | 62.9 | 35 | 23 |  | 63.9 | 48 | 21 |
|  | Mann-Whitney U test | *U* = 133.5; *p* = 0.130 | | |  | *U* = 131.5; *p* = 0.169 | | |  | *U* = 101.5; *p* = 0.730 | | |  | *U* = 396.5; *p* = 0.855 | | |
| PANSS_0-6_ positive symptom scores reduction after 8 weeks of treatment | | | | | | | | | | | | | | | | |
| *COMT* rs4680-rs4818 | |  |  |  |  |  |  |  |  |  |  |  |  |  |  |  |
|  | C-A carriers | 75.0 | 79 | 34 |  | 63.3 | 86 | 33 |  | 81.2 | 77 | 32 |  | 73.7 | 78 | 30 |
|  | Non-carriers | 52.9 | 62 | 43 |  | 71.9 | 32 | 17 |  | 66.7 | 49 | 28 |  | 77.8 | 62 | 35 |
|  | Mann-Whitney U test | *U* = 128.0; *p* = 0.100 | | |  | *U* = 134.5; *p* = 0.131 | | |  | *U* = 97.5; *p* = 0.611 | | |  | *U* = 435.5; *p* = 0.680 | | |
| PANSS_0-6_ negative symptom scores reduction after 8 weeks of treatment | | | | | | | | | | | | | | | | |
| *COMT* rs4680-rs4818 | |  |  |  |  |  |  |  |  |  |  |  |  |  |  |  |
|  | C-A carriers | 38.9 | 86 | 34 |  | 64.1 | 80 | 44 |  | 35.4 | 73 | 39 |  | 60.0 | 88 | 34 |
|  | Non-carriers | 38.9 | 57 | 20 |  | 69.2 | 53 | 26 |  | 40.0 | 58 | 35 |  | 53.3 | 66 | 33 |
|  | Mann-Whitney U test | *U* = 172.5; *p* = 0.611 | | |  | *U* = 123.5; *p* = 0.307 | | |  | *U* = 127.5; *p* = 0.476 | | |  | *U* = 393.0; *p* = 0.813 | | |
| PANSS_0-6_ general psychopathology scores reduction after 8 weeks of treatment | | | | | | | | | | | | | | | | |
| *COMT* rs4680-rs4818 | |  |  |  |  |  |  |  |  |  |  |  |  |  |  |  |
|  | C-A carriers | 48.7 | 83 | 26 |  | 48.6 | 77 | 23 |  | 55.8 | 62 | 34 |  | 56.7 | 92 | 30 |
|  | Non-carriers | 45.8 | 58 | 11 |  | 58.6 | 47 | 24 |  | 44.6 | 41 | 31 |  | 51.1 | 89 | 21 |
|  | Mann-Whitney U test | *U* = 152.0; *p* = 0.308 | | |  | *U* = 141.5; *p* = 0.067 | | |  | *U* = 96.0; *p* = 0.583 | | |  | *U* = 388.5; *p* = 0.760 | | |

Values are given as median. range and interquartile range (IR); PANSS = Positive and Negative Syndrome Scale.
